# Recent advances in 2D, 3D and higher-order topological photonics

**DOI:** 10.1038/s41377-020-0331-y

**Published:** 2020-07-20

**Authors:** Minkyung Kim, Zubin Jacob, Junsuk Rho

**Affiliations:** 1 Department of Mechanical Engineering, Pohang University of Science and Technology (POSTECH), Pohang, 37673 Republic of Korea; 2 School of Electrical and Computer Engineering, Birck Nanotechnology Center, Purdue University, West Lafayette, IN 47906 USA; 3 Department of Chemical Engineering, Pohang University of Science and Technology (POSTECH), Pohang, 37673 Republic of Korea

**Keywords:** Metamaterials, Nanophotonics and plasmonics, Photonic devices, Sub-wavelength optics

## Abstract

Over the past decade, topology has emerged as a major branch in broad areas of physics, from atomic lattices to condensed matter. In particular, topology has received significant attention in photonics because light waves can serve as a platform to investigate nontrivial bulk and edge physics with the aid of carefully engineered photonic crystals and metamaterials. Simultaneously, photonics provides enriched physics that arises from spin-1 vectorial electromagnetic fields. Here, we review recent progress in the growing field of topological photonics in three parts. The first part is dedicated to the basics of topological band theory and introduces various two-dimensional topological phases. The second part reviews three-dimensional topological phases and numerous approaches to achieve them in photonics. Last, we present recently emerging fields in topological photonics that have not yet been reviewed. This part includes topological degeneracies in nonzero dimensions, unidirectional Maxwellian spin waves, higher-order photonic topological phases, and stacking of photonic crystals to attain layer pseudospin. In addition to the various approaches for realizing photonic topological phases, we also discuss the interaction between light and topological matter and the efforts towards practical applications of topological photonics.

## Topological photonics: Light and topology linked in theory and practice

The structural property of materials known as their topology can be exploited to control the behavior of light in the emerging field of topological photonics, offering potential applications in areas including topology theory, optical communication and optical computing. Researchers in South Korea and the USA, led by Junsuk Rho at Pohang University of Science and Technology (POSTECH) in South Korea, review recent progress in this rapidly developing field. They consider detailed theoretical and practical aspects of the interaction of light with materials whose topology influences the behavior of light waves. These materials include ‘photonic crystals’ and ‘metamaterials’, which have structural features on scales shorter than the wavelength of light. The authors also discuss progress towards developing practical applications for topological photonics. The ability to achieve highly efficient light transmission without dissipation is particularly promising.

## Introduction

**Fig. 1:**
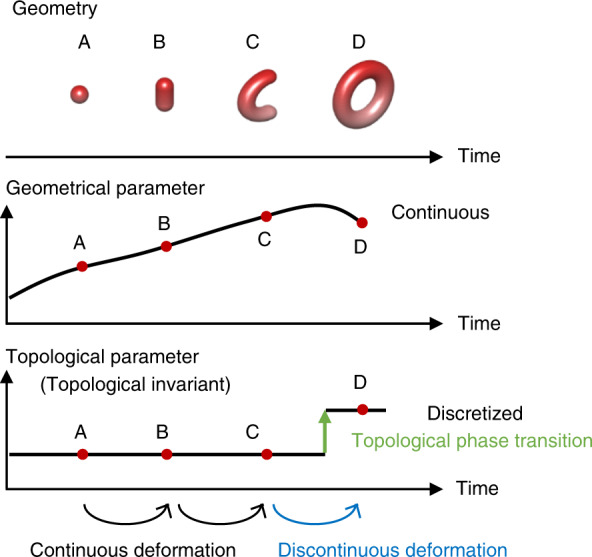
Illustration of geometrical and topological parameters changing over time. When a sphere transforms to a torus by elongation and end-to-end connection, geometrical parameters, such as the surface-to-volume ratio, continuously change, while a topological parameter, called a topological invariant, undergoes an abrupt and discontinuous change. A deformation that does not alter the topological invariant is called a continuous deformation, and a deformation that changes the topological invariant is called a discontinuous deformation. The transition between topologically inequivalent states is called a topological phase transition

Topology is a field of mathematics that studies conserved and quantized quantities, which are known as topological invariants. Two objects that have the same topological invariants are defined as topologically equivalent. For instance, imagine a sphere that transforms via two intermediates to a torus in time (Fig. 1a–d). Whereas the geometrical parameters continuously change in time, a topological parameter, or a topological invariant, is discretized as an integer. We consider the number of holes on a surface as a topological invariant; it is zero for objects A to C and unity for object D. Deformations from A to B and from B to C preserve the topological invariant and are called continuous because the parameters continuously change during the deformation. Therefore, objects A, B, and C can be transformed into each other by a continuous deformation, irrespective of its strength. More generally, any two topologically equivalent objects can be transformed into each other by an arbitrary continuous deformation, such as stretching and compression. In contrast, deformations that involve cutting, tearing, or attachment alter topological invariants. These deformations are called discontinuous in that a topological invariant abruptly changes at a certain moment. The deformation from C to D belongs to this case because the topological invariant changes from zero to unity. The transition between topologically distinct states is called a topological phase transition.

The definition of topological equivalence implies that if a physical phenomenon is characterized by a topological invariant, then the phenomenon does not change under any continuous deformation. This perspective is fundamentally different from the previous understanding that phases of matter can be described by spontaneous symmetry breaking. This robust feature of topology has spawned a new paradigm called topological order^1^ and has explained many experimental observations that could not be described by other concepts. A good example is the quantum Hall effect^2^, in which the Hall conductance of a 2D electron gas under an external magnetic field has a quantized value with remarkable precision regardless of the charge density and impurities in the sample. This extraordinary behavior cannot be explained by Landau’s local-order parameters^3^ but can be understood by topological order.

Topology is becoming a universal notion throughout physics. Starting from condensed matter physics^4^, the concept of topology has been extended to various systems, including photonics, phononics^5^, mechanics^6^, and cold atomic gases^7^. In particular, the implementation of topology in photonics has been motivated by three reasons. First, photonics provides a concrete and versatile platform for realizing and exploring topological features because the photon wavelength is much longer than the electron wavelength. Photons have wavelengths on the order of hundreds of nanometers for visible light and larger for lower frequencies, whereas electrons with 1 eV energy have wavelengths on the order of nanometers. Therefore, without going down to the atomic level, the photonic band structure and its topology can be engineered by appropriately artificially structuring and arranging the unit design on a scale comparable to or smaller than the wavelength. The booming interest in light manipulation over the past couple of decades using photonic crystals and metamaterials has facilitated topological photonic band engineering. Indeed, despite the fundamental difference between fermionic and bosonic systems, many extraordinary features that have been predicted in condensed matter physics or high-energy physics have been found in photonics. Second, consisting of two vector fields, electromagnetic waves enable richer physics compared to other classical scalar waves. For example, electromagnetic duality can be used to emulate electronic spin states^8^, and the vectorial nature of electric fields gives rise to the definition of polarization and to resultant properties such as chirality^9^. Last, adding topology to photonics facilitates robust control of photons on a wavelength scale. Current communication technologies use electrons as information carriers. However, compared to electrons, photons are faster, dissipate less, and support more channels. However, because the propagation of photons is sensitive to the surface at a scale comparable to the wavelength, impurities and defects at the surface easily scatter photons. Therefore, optical communication relies on guiding based on the refractive index, such as in optical fibers, which makes the whole system bulky. Topology can provide a solution to this practical problem by providing a robust way to control photons in compact systems. The application of topology to photonics may yield many practical applications, such as compact waveguides with no bending loss, lasers, and cavities.

During the past several years, there has been vast progress in topological photonics. The emerging fields of topological photonics have been reviewed many times^10–16^. A comprehensive review^13^ exists that covers most of the progress in topological photonics in a broad aspect, in addition to some reviews focused on specific topics, such as two-dimensional (2D) topological photonics^12,16^, topological nanophotonics^15^, active topological photonics^17^, nonlinear topological photonics^18^, and topological photonics using synthetic dimensions^14^. However, approaches to three-dimensional (3D) photonic topological phases have been considered only as a minor part of these reviews. Additionally, following the rapid progress in topological photonics, a review that covers the latest achievements is in demand. Therefore, this review is devoted to 3D topological phases and their photonic realization and to the latest progress in this rapidly evolving field.

In the first part, we review topological band theory and three 2D topological phases: the quantum Hall, quantum spin Hall, and quantum valley Hall phases. The basics of topological band theory and some representative topological phases have been previously reviewed^4,19,20^. However, they are mostly described in the context of condensed matter physics and are difficult to follow for general readers in photonics who do not have a firm background in this field. Thus, we concisely review the basics of topological band theory and some 2D topological phases, especially for readers who are interested in topological photonics but are not familiar with condensed matter physics. Before finishing this section, we briefly cover other topological phases, such as the Floquet topological phase and Zak phase.

Then, we cover 3D photonic topological phases and various approaches for realizing them. We aim to discuss photonic systems with 3D topological phases and especially focus on approaches that use photonic crystals or metamaterials. Various symmetry breakings and material responses to achieve topological phases are elucidated. The symmetries referred to in this review are visualized in Table 1. Five symmetries, time-reversal symmetry (${\cal{T}}$), inversion symmetry (${\cal{P}}$), glide reflection symmetry, screw symmetry and electromagnetic duality, are discussed. ${\cal{T}}$ refers to a symmetry under time-reversal operation^21^ (*t* *→* −*t*). ${\cal{P}}$, glide reflection symmetry and screw symmetry are spatial symmetries related to symmetries under spatial transformation^21,22^. Electromagnetic duality is associated with a symmetry between electric and magnetic fields and is an intrinsic property of the Maxwell equations in vacuum^23^. Not only symmetries but also material responses can be exploited to realize topological phases. Five material responses, gyrotropic^24^, bianisotropic^25^, chiral^26^, hyperbolic^27^, and double hyperbolic^28^ responses, the constitutive equations of which are shown in Table 2, will be covered.

**Table 1:** Overview of symmetries discussed in this review

Symmetries	Operation
Time-reversal symmetry^21^ (${\cal{T}}$)	*t* → −*t****, x*** → ***x***, ***E*** → ***E***, ***H*** → −***H***
Inversion symmetry^21^ (or parity symmetry, ${\cal{P}}$)	***x*** → **−*x,*** *t* → *t*, ***E*** → **−*E***, ***H*** → ***H***
Glide reflection symmetry^22^	Reflection + translation $\hat G_{\mathrm{x}}\!:\left( {x,y,z} \right) \to \left( {1{\mathrm{/}}2 - x,1{\mathrm{/}}2 + y,z} \right)$
Screw symmetry^22^	Rotation + translation along the rotational axis $\hat S_{\mathrm{x}}\!:\left( {x,y,z} \right) \to \left( {x + 1{\mathrm{/}}2, - y, - z} \right)$
Electromagnetic duality^23^	***E*** → −*c****B***, *c****B*** → ***E,*** *ε* = *αμ* (*α* is a constant scalar)

The bold values indicate that the quantity is a vector

**Table 2:** Overview of material responses discussed in this review

Material responses	Constitutive equations
Gyrotropic^24^	Gyroelectric $${\boldsymbol{D}}=\left({\begin{array}{*{20}{c}}{\varepsilon_1}&{i\varepsilon_2}&0\\{-i\varepsilon_2}&{\varepsilon_1}&0\\0&0&{\varepsilon_3}\end{array}}\right){\boldsymbol{E}}$$, Gryomagnetic $${\boldsymbol{B}} = \left( {\begin{array}{*{20}{c}} {\mu _1} & {i\mu _2} & 0 \\ { - i\mu _2} & {\mu _1} & 0 \\ 0 & 0 & {\mu _3} \end{array}} \right){\boldsymbol{H}}$$
Bianisotropic^25^ (magneto-electric coupling)	$${\boldsymbol{D}} = \varepsilon {\boldsymbol{E}} + \left( {\begin{array}{*{20}{c}} 0 & {i\chi } & 0 \\ { - i\chi } & 0 & 0 \\ 0 & 0 & 0 \end{array}} \right){\boldsymbol{H}},\,{\boldsymbol{B}} = \left( {\begin{array}{*{20}{c}} 0 & {i\chi } & 0 \\ { - i\chi } & 0 & 0 \\ 0 & 0 & 0 \end{array}} \right){\boldsymbol{E}} + \mu {\boldsymbol{H}}$$
Chiral^26^	$${\boldsymbol{D}} = \varepsilon {\boldsymbol{E}} + \left( {\begin{array}{*{20}{c}} {i\kappa } & 0 & 0 \\ 0 & {i\kappa } & 0 \\ 0 & 0 & {i\kappa } \end{array}} \right){\boldsymbol{H}},\,{\boldsymbol{B}} = - \left( {\begin{array}{*{20}{c}} {i\kappa } & 0 & 0 \\ 0 & {i\kappa } & 0 \\ 0 & 0 & {i\kappa } \end{array}} \right){\boldsymbol{E}} + \mu {\boldsymbol{H}}$$
Hyperbolic^27^	$${\boldsymbol{D}} = \left( {\begin{array}{*{20}{c}} {\varepsilon _{\mathrm{x}}} & 0 & 0 \\ 0 & {\varepsilon _{\mathrm{y}}} & 0 \\ 0 & 0 & {\varepsilon _{\mathrm{z}}} \end{array}} \right){\boldsymbol{E}},\,{\boldsymbol{B}} = \mu {\boldsymbol{H}}$$ One or two components among *ε*_x_, *ε*_y_, and *ε*_z_ have the opposite sign.
Double hyperbolic^28^	$${\boldsymbol{D}} = \left( {\begin{array}{*{20}{c}} {\varepsilon _{\mathrm{x}}} & 0 & 0 \\ 0 & {\varepsilon _{\mathrm{y}}} & 0 \\ 0 & 0 & {\varepsilon _{\mathrm{z}}} \end{array}} \right){\boldsymbol{E}},\,{\boldsymbol{B}} = \left( {\begin{array}{*{20}{c}} {\mu _{\mathrm{x}}} & 0 & 0 \\ 0 & {\mu _{\mathrm{y}}} & 0 \\ 0 & 0 & {\mu _{\mathrm{z}}} \end{array}} \right){\boldsymbol{H}}$$ One or two components among *ε*_x_, *ε*_y_, and *ε*_z_ have the opposite sign, and one or two components among *μ*_x_, *μ*_y_, and *μ*_z_ have the opposite sign.

The bold values indicate that the quantity is a vector

In the last part, to follow the rapid development of topological photonics, some recently emerging fields that have not hitherto been discussed by previous reviews are focused on. More specifically, this part includes six subtopics: stacked topological photonic crystals for layer pseudospin, Weyl degeneracies in nonzero dimensions, unidirectional Maxwellian spin waves, higher-order photonic topological phases, interactions between light and topological phases and progress towards application of topological photonics. We conclude this review by providing a perspective on this promising field.

## Topological band theory

Conventional band theory in photonics has been developed for photonic crystals^29–31^. Similar to electrons propagating through a crystal, photons in a photonic crystal experience a periodic potential, and the global features can be delineated by the photonic band structure^32^. The photonic band dispersion, which has been generally discussed in terms of the photonic band gap, has another interesting characteristic called topology. The topology originates from winding of eigenmodes in momentum space and can be characterized by a topological invariant. Apart from the band dispersion, each band has a topological invariant that is zero for topologically trivial cases and nonzero for topologically nontrivial cases. Two bands are defined to be topologically equivalent if they can be adiabatically transformed into each other^33^. Therefore, two topologically equivalent insulators are connected by a continuous deformation, along which a band gap remains open. In contrast, a topological insulator and an ordinary insulator cannot be adiabatically transformed into each other. Instead, they can only be interconverted if the band gap is closed and reopened^33^. The band gap closing corresponds to cutting of the photonic band dispersion in momentum space and alters the bulk topology. This topological phase transition ensures gapless boundary modes at the interfaces between a topological insulator and an ordinary insulator. The existence of these edge modes across the band gap is consistent with the definition of topological equivalence of bands and is closely related to the bulk topology. Indeed, the number of gapless edge modes is equal to the difference between the topological invariants of the two media^34,35^. This so-called bulk-boundary correspondence^34,35^ dictates that the number of lower-dimensional modes, or boundary modes, is determined by the topology of the bulk states. Because the existence of the gapless boundary modes is protected by the bulk topology, these modes provide robust characteristics insensitive to small perturbations that do not change topology.

**Fig. 2:**
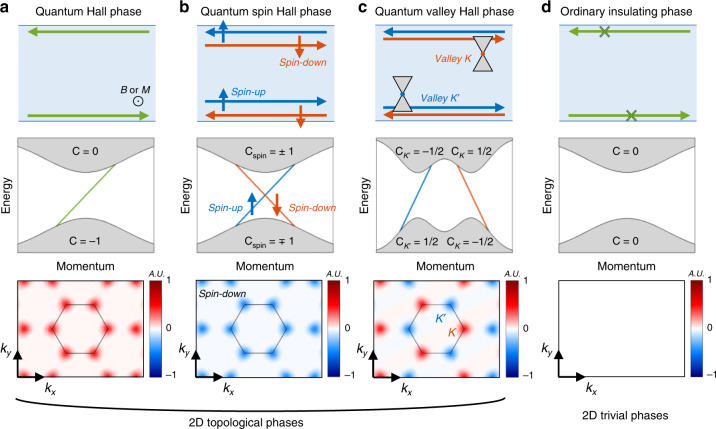
Schematic illustrations of the edge states and Berry curvature of the **a** quantum Hall phase, **b** quantum spin Hall phase, **c** quantum valley Hall phase, and **d** ordinary insulating phase. The hexagons in the bottom row denote the first Brillouin zone (BZ). **a** Unidirectional propagation of edge states along the boundary in the quantum Hall phase. The edge state dispersion has a fixed sign of the group velocity. The Berry curvature is nonzero. **b** Spin-dependent unidirectional propagation of edge states along the boundary in the quantum spin Hall phase. The edge state dispersion shows a spin-locked group velocity. The Berry curvature is positive for spin-up and negative for spin-down, showing a nonzero spin Chern number but a zero total Chern number. The Berry curvature of the spin-down mode is plotted. **c** Valley-dependent unidirectional propagation of edge states along the boundary in the quantum valley Hall phase. The edge state dispersion has a valley-locked group velocity. The Berry curvature has positive and negative hot spots at the valleys. The valley Chern number, which can be calculated by integrating the Berry curvature over half of the BZ, is nonzero. **d** The ordinary insulating phase supports neither conducting edge states nor a nonzero Berry curvature. Green line: edge states in the quantum Hall phase; blue and red lines: edge states with positive and negative group velocities, respectively

In this section, we discuss a few representative 2D topological phases by classifying them according to their topological invariants. Three topological phases—the quantum Hall phase, quantum spin Hall phase, and quantum valley Hall phase—are reviewed (Fig. 2a–c) and compared to the ordinary insulating phase (Fig. 2d). A brief and general discussion on other topological phases then follows. Since the details of such topological phases^4,19,20^ and attempts in photonics to emulate them^10,12,13^ can be found elsewhere in the literature, we do not go into detail but discuss important features and conditions so that readers who are not familiar with topology can grasp the concept and appreciate the rest of the paper.

### Quantum Hall phase

The discovery of the quantum Hall phase goes back to 1980 when Klitzing, Dorda, and Pepper observed that a 2D electron gas under a perpendicular magnetic field has a quantized Hall conductance^2^. This effect corresponds to the quantum version of the conventional Hall effect, in which electrons in a metal plate under an applied magnetic field are deflected by the Lorentz force. In contrast to the Hall effect, the strong magnetic field and cryogenic conditions in the quantum Hall state accentuate the quantized feature of the Hall conductance. It was later revealed that a magnetic flux is not a necessary condition for such quantum Hall effects^19^. Other mechanisms, such as magnetization and strong spin–orbit coupling in magnetic materials that break ${\cal{T}}$, can induce a similar effect in the absence of a magnetic field^19^.

The signature of the quantum Hall phase is that electrons are localized in the bulk but unidirectionally propagate along the boundaries (Fig. 2a, top row). The broken ${\cal{T}}$ makes the propagation of edge modes chiral. In other words, electrons propagate either counterclockwise or clockwise. Propagation in the reverse direction is not allowed, as implied by the fixed sign of the group velocity (Fig. 2a, middle row). Therefore, the edge states are not back-scattered, even in the presence of large defects. A topological invariant of the quantum Hall phase is the integer of the Hall conductance, which is called the Chern number (or TKNN invariant)^1,36^

1 $$C_{\mathrm{n}} = \frac{1}{{2\pi }}{\int\nolimits_{{\mathrm{BZ}}}} {{\boldsymbol{F}}_{\mathrm{n}}d^2{\boldsymbol{k}}}$$

where BZ denotes the Brillouin zone, ${\boldsymbol{F}}_{\mathrm{n}} = \nabla \times i{\boldsymbol{u}}_{\mathrm{n}}({\boldsymbol{k}})|\nabla _{\boldsymbol{k}}|{\boldsymbol{u}}_{\mathrm{n}}({\boldsymbol{k}})$ is the Berry curvature of the *n*-th band, and $u_{\rm n}$ is the *n*-th eigenstate^37^. An example of the Berry curvature of a system possessing the quantum Hall phase is shown in Fig. 2a, bottom row. The Chern number of a medium that possesses a quantum Hall phase is nonzero.

It has been suggested that photons in 2D photonic crystals composed of magnetoelectric materials behave like electrons under the quantum Hall effect^38,39^. The magnetoelectric coupling breaks ${\cal{T}}$, resulting in a nonzero Chern number across a photonic band gap. At the band gap, chiral edge transport immune from back-scattering was theoretically predicted^39^. Gyromagnetic photonic crystals, which exhibit strong gyromagnetic anisotropy, have been proposed as a realistic platform to manifest the photonic analog of the quantum Hall effect^40^. This theoretical prediction was soon experimentally confirmed in the microwave regime by showing chiral edge propagation robust against an artificially introduced scatterer^41^. The quantum Hall phase has also been demonstrated in photonics using Floquet-like waveguide arrays^42^, dynamic modulation^43^, magnetoplasmons^44^, and graphene^45^.

### Quantum spin Hall phase

Difficulties in applying magnetic fields and the scarcity of gyromaterials make it difficult to realize the quantum Hall effect. Challenges in breaking ${\cal{T}}$ have motivated the search for topological phases in ${\cal{T}}$-invariant systems. Spin, a fundamental characteristic of electrons, provides a solution. According to Kramers’ theorem^46^, all eigenstates of a system that possesses half-integer spin are at least doubly degenerate under ${\cal{T}}$. Because electrons with spin-up experience different forces than electrons with spin-down in the presence of spin–orbit coupling, the two classes of electrons may have different topological behaviors despite the degenerate energy level. In other words, electrons with spin-up may propagate along the boundaries counterclockwise while electrons with spin-down propagate clockwise, or vice versa (Fig. 2b, top row). At the band gap of a quantum spin Hall insulator, gapless edge states for each spin exist, and the sign of the group velocity of the edge states is locked by the spin (Fig. 2b, middle row). This spin-momentum-locked characteristic^47,48^ enables topologically protected helical edge states that propagate without back-scattering if the spin is not flipped.

This quantum spin Hall state can be viewed as two copies of the quantum Hall state for each spin. The Berry curvature of the quantum spin Hall phase is spin-dependent. For example, the Berry curvature of electrons with spin-up is positive, and that of electrons with spin-down is negative (Fig. 2b, bottom row). Thus, the Chern number calculated for a specific spin has a nonzero value under ${\cal{T}}$ (*C*_↑_ = − *C*_↓_ ≠ 0, where *C*_↑_ is the Chern number of spin-up electrons and *C*_↓_ is the Chern number of spin-down electrons), although the Chern number of the two spins cancel each other, leading to a zero Chern number (*C* = *C*_↑_ + *C*_↓_ = 0). This is a relaxed condition compared to the quantum Hall phase, considering that a nonzero spin Chern number *C*_spin_ = (*C*_↑_ − *C*_↓_)/2 instead of a nonzero Chern number suffices. The quantum spin Hall phase is characterized by the ${\Bbb{Z_{2}}}$ invariant, which is associated with the spin Chern number by^4,49,50^ *C*_spin_(mod 2). The *Z*_2_ invariant has only two integer values: 0 for topologically trivial cases and 1 for nontrivial cases.

At a glance, the quantum spin Hall phase seems incompatible with photonics. Kramers’ theorem is not applicable to photons because they do not have half-integer spins. However, photons, even in free space, have spin properties as a result of circular polarization^9,51^. The spin-locked characteristics of boundary modes have been studied for 2D interfaces between vacuum and a metal^9^, between dielectric media, between a metal and a dielectric^47^, and between definite and indefinite anisotropic media^52^. Additionally, photons inside structured matter can have a spin-like quantity called pseudospin by using electromagnetic duality^8^ or crystalline symmetries^53^ to emulate the fermionic ${\cal{T}}$ operator ${\cal{T}}_{\mathrm{f}}^2 = - 1$. The photonic analog of the quantum spin Hall phase has been actively investigated using a 2D array of resonators^54,55^ and photonic crystals that possess crystalline symmetries^53,56,57^. In the latter instance, a quantum spin Hall phase of a perturbed photonic crystal has an integer *C*_spin_ only in the limit of zero-order perturbation theory^58^. Beyond this limit, *C*_spin_ may depend on the strength of the perturbation and cannot serve as a topological invariant.

### Quantum valley Hall phase

In the previous section, we reviewed the quantum spin Hall phase, where the spin degree of freedom guarantees spin-polarized topological edge states. Similarly, there exists another binary degree of freedom: a valley has recently been identified as a candidate to yield a new topological phase under ${\cal{T}}$, the so-called quantum valley Hall phase. A valley refers to momentum with a local energy extremum. When a Dirac point at a valley is lifted by symmetry breaking, the band gap may support the quantum valley Hall phase. In a quantum valley Hall system, electrons at different valleys (*K* and *K*′) propagate along the boundaries in the opposite directions (Fig. 2c, top row). The edge state dispersion of a quantum valley Hall insulator exhibits opposite signs of the group velocity (Fig. 2c, middle row).

Breaking of ${\cal{P}}$ is a prerequisite of the quantum valley Hall phase. This condition can be easily understood by symmetry consideration of Berry curvatures. The Berry curvature of the *n*-th band satisfies ***F***_n_(***k***) = −***F***_n_(−***k***) under ${\cal{T}}$ and ***F***_n_(***k***) = ***F***_n_(−***k***) under ${\cal{P}}$. Therefore, the Berry curvature is zero in the whole BZ in a system that preserves both ${\cal{T}}$ and ${\cal{P}}$. However, in a system that lacks ${\cal{P}}$, the Berry curvature can have a nonzero value localized at valleys (Fig. 2c, bottom row). The Berry curvature at the two valleys *K* and *K*′ have opposite signs, which leads to a total Chern number of zero. The topological invariant of the quantum valley Hall effect is *C*_valley_, which can be calculated by replacing the BZ in Eq. (1) with the half of the BZ around the valley. Along the interfaces between media with distinct *C*_valley_, this valley-momentum locking enables edge transport robust against perturbations that do not cause intervalley scattering^59^.

Despite the relatively short history compared to that of the quantum Hall and quantum spin Hall effects, the quantum valley Hall effect has been intensively studied. Before finishing this section, we briefly introduce recent work on the quantum valley Hall phase in photonics. The quantum valley Hall effect in photonics was first proposed in 2016 by breaking the ${\cal{P}}$ of triangularly arranged dielectric rods^59^. Before ${\cal{P}}$ is broken, the photonic crystal consists of circular rods arranged in a hexagonal lattice, in which the spatial symmetry gives rise to a Dirac point at valleys. Application of a ${\cal{P}}$-breaking perturbation by changing the cross-sectional shape of the rods from circles to triangles suppresses the intervalley scattering, which lifts the Dirac point and opens a complete band gap. The authors numerically demonstrated unidirectional propagation of edge states and efficient in-coupling and out-coupling^59^. The quantum valley Hall effect and valley-dependent robust propagation have been experimentally observed in microwaves using designer surface plasmon crystals^60^. Studies on valley-dependent transport were further extended to surface waves that propagate in a specified direction determined by their spins inside a bulk photonic crystal^61^. In this case, ${\cal{P}}$ is broken by differentiating the heights of the two sublattices of the honeycomb lattice. The quantum valley Hall effect has been confirmed numerically by using dielectric rods in a perturbed kagome lattice^62^ and experimentally by using coupled waveguide arrays with detuned refractive indices^63^, a dielectric slab with air holes with a ${\cal{P}}$-breaking shape^64,65^ and ${\cal{P}}$-broken connected dielectrics that have dual-band edge states^66^. The quantum valley Hall phase can also be implemented in an active system. Recently, topologically protected lasing modes were demonstrated by combining the quantum valley Hall effect with an electrically pumped laser^67^.

A photonic crystal can simultaneously possess both valley-dependent and spin-dependent properties. A hexagonal array of staggered bianisotropic rods has a bulk dispersion, for which the spin-dependent features appear in a valley. The interaction between the valley and spin enables valley-selective net spin flow inside bulk photonic crystals^68^. Additionally, the quantum spin and valley Hall phases can be independently controlled in a photonic crystal. In such a system, two perturbations, one to induce a quantum spin Hall phase by adding bianisotropy and the other to induce a quantum valley Hall phase by breaking ${\cal{P}}$, are present. The topological phase of the system is determined by the relative strengths of the two perturbations. By varying the strengths of the two perturbations, a topological phase transition between the quantum spin and valley Hall phases was demonstrated. This work suggested a photonic system in which the topological phase can be controlled by both the spin and valley degrees of freedom^69^.

### Other topological phases

In addition to the aforementioned three topological phases, there are other topological phases that originate from different mechanisms. One example is the Floquet topological phase, which can be understood as a time counterpart of the Bloch theorem. Floquet’s theorem states that a solution of a time-periodic Hamiltonian can be expressed by multiplying a time-periodic function by a phase term^70^. This theorem has been used to study periodically driven quantum systems. Interesting phenomena occur when we examine the results of the time periodicity. As a translational symmetry originating from a potential periodic in space gives rise to conservation of quasi-momentum^71^ (crystal momentum), a potential periodic in time results in conservation of a quantity according to Noether’s theorem^72^. This conserved quantity is a quasi-energy and can serve as an additional dimension along with crystal momenta. Therefore, similar to how spatially periodic structures can possess a topological phase, a system under periodic temporal modulation can also support a topological phase called the Floquet topological phase^73–76^. This phenomenon has also been realized in photonics by using time-dependent index modulation^77,78^ and by using the propagation axis of a waveguide array to mimic a time-like axis^42,79,80^.

Another topological phase is called the Zak phase, along with its 2D version^81,82^. The Zak phase is obtained by integrating the Berry connection along one wave vector axis and can be viewed as a 1D version of the Berry phase. The topology of the Zak phase originates in bulk polarization, or a shift of the Wannier band, and can have only two values: 0 for trivial topology and π for nontrivial topology^81^. The edge modes in a 2D system that carry the nontrivial Zak phase are floating in the band gap and not connected to the bulk bands. Additionally, the topological protection of the Zak phase is weak compared to the previously mentioned topological phases^82^.

## Three-dimensional photonic topological phases

### 3D gapped phase

**Fig. 3:**
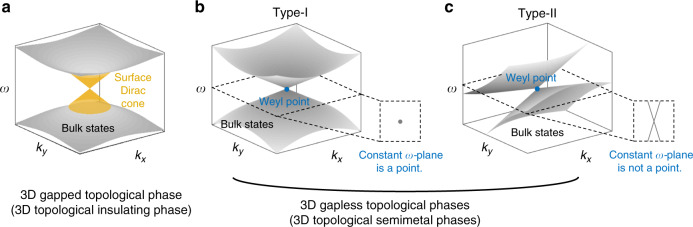
Dispersions of 3D topological phases. **a** 3D gapped topological phase (or 3D topological insulating phase) with a surface Dirac cone (yellow). **b**, **c** 3D gapless topological phases (or 3D topological semimetal phases) hosting **b** a type-I and **c** a type-II Weyl point (blue). The insets show the equi-frequency curves at the frequency of the Weyl point. Only the *k*_x_ and *k*_y_ axes among the three momenta are drawn due to the limited dimensions

In the previous section, the topological phases of 2D insulators were reviewed. Such systems are generally composed of unit cells periodically arranged along two axes, and the resultant two crystal momenta form the 2D BZ. The gapped 2D bulk states support gapless edge states with topological protection. The 2D topological insulating phases can be further extended to 3D by using a 3D crystalline structure that consists of unit cells periodically arranged along all three spatial directions. These 3D gapless topological phases, also known as 3D topological insulating phases, are attributed to the 3D band gap that separates topologically inequivalent bands. If two 3D bulk bands, one of which is above the band gap and the other below it, are topologically distinct, then gapless surface states appear inside the band gap^83–85^ (Fig. 3a) as 2D gapped bulk states host gapless edge states in 2D topological phases. The edge states of 2D topological insulators are confined in a 2D plane, so their propagations are protected by topology only along the planar directions. In contrast, the surface states of 3D topological insulators are confined in all three spatial directions^86^. In other words, a 3D photonic gapped phase enables photons to propagate robustly against defects along any spatial direction without being limited to a certain plane.

3D gapped topological phases can be realized in both ${\cal{T}}$-broken and ${\cal{T}}$-invariant systems. The 3D quantum Hall phase, which can be regarded as a 3D extended version of the 2D quantum Hall phase, corresponds to the ${\cal{T}}$-broken case. The Chern number, the topological invariant of the 2D quantum Hall phase, is defined only in even spatial dimensions. In 3D cases, the quantum Hall phase is characterized by a triad of Chern numbers: **C**^(1)^ ≡ (*C*_x_^(1)^, *C*_y_^(1)^, *C*_z_^(1)^), where *C*_j_^(1)^ is the Chern number of the 2D momentum plane normal to the *j*-axis. The superscript (1) is added to distinguish the Chern numbers from the second Chern number^87^, which characterizes the quantum Hall phases in four dimensions^88^.

The 3D quantum Hall phase has been theoretically predicted by examining 3D electron gases under magnetic fields^89–91^ and has been experimentally demonstrated using semiconductor superlattices constructed by stacking 2D quantum Hall insulators with appropriate interlayer coupling^92^. However, the 3D quantum Hall phase in a strict sense, with surface states closed along the stacking direction, was only recently observed^93^. A quantized Hall resistivity and conductive surface states were confirmed in ZrTe_5_ crystals under magnetic fields. A photonic analog of the 3D quantum Hall phase was theoretically proposed by using photonic crystals composed of gyroelectric materials^94^. A magnetic field bias breaks ${\cal{T}}$ and produces a band gap and topological surface states within the gap.

Similarly, the 3D quantum spin Hall phase can be viewed as a generalization of the 2D quantum spin Hall phase and corresponds to the ${\cal{T}}$-invariant case. The 3D quantum spin Hall phase is characterized by four ${\Bbb{Z_{2}}}$ invariants^83–85^ (*ν*_0_; *ν*_1_ *ν*_2_ *ν*_3_). To understand these invariants, we imagine a *k*_x_ *−* *k*_y_ plane by fixing the *k*_z_ of a given 3D BZ. A ${\Bbb{Z_{2}}}$ invariant can be defined in only two momentum planes, *k*_z_ = 0 and *k*_z_ = *π/a*, where *a* is a lattice constant, as they are the only ${\cal{T}}$-symmetric planes. If the ${\Bbb{Z_{2}}}$ invariants of the two planes are distinct, then the ${\Bbb{Z_{2}}}$ invariants of the two planes defined by the other two directions should also be different^85^. This change in ${\Bbb{Z_{2}}}$ invariants between two planes is described by the strong topological invariant *ν*_0_; if one of the planes has ${\Bbb{Z_{2}}}$ = 0 and the other has ${\Bbb{Z_{2}}}$ = 1, then the system is topologically nontrivial and has *ν*_0_ = 1. In contrast, if the ${\Bbb{Z_{2}}}$ invariants of the two planes are the same, then the system has *ν*_0_ = 0 and is trivial. The other three invariants are weak invariants, each of which corresponds to the ${\Bbb{Z_{2}}}$ invariant of *k*_i_ = *π/a* (*i* = {*x, y, z*})^83^.

*ν*_0_ classifies 3D quantum spin Hall insulators into *weak* topological insulators (*ν*_0_ = 0) and strong topological insulators^4,33^ (*ν*_0_ = 1). Weak topological insulators can be achieved by layering 2D quantum spin Hall insulators similar to how 3D quantum Hall insulators are formed by stacking 2D quantum Hall insulators. For weak topological insulators, the ${\Bbb{Z_{2}}}$ invariants of the two 2D planes (*k*_z_ = 0 and *k*_z_ = *π/a*) are the same, so only an even number of surface Dirac cones are possible^4,33^. On the other hand, strong topological insulators require other mechanisms, such as strong spin–orbit coupling, and support an odd number of surface Dirac cones^95,96^. In 2011, the first proposal of a 3D topological insulating phase in photonics was reported^97^. Following the scheme of topological crystalline insulators in an electronic system^98^, a tetragonal lattice of uniaxial dielectric cavities in a lossless metallic host was investigated using a coupled dipole method. This system with ${\cal{T}}$ and point-group symmetry exhibits a complete 3D band gap and gapless topological surface states. Topological photonic systems with 3D band gaps have also been proposed by using 3D bianisotropic structures^86,99,100^ and a 2D crystalline structure with a synthetic dimension^78^.

### 3D gapless phase

The 3D gapped phase is not the only type of phase possible among 3D topological phases. Discovery of a 3D gapless bulk dispersion that hosts nontrivial band topology^101–106^ refuted the perception that topological features necessarily require a band gap and has extended our understanding of the topological phases. The 3D gapless topological phase, also known as the topological semimetal phase, is a new topological phase of matter different from the topological insulating phase (Fig. 3b, c). Unlike 3D gapped phases, the 3D gapless phase has no 2D counterpart. Instead of a band gap, the 3D gapless topological phases are characterized by Weyl degeneracies, which are degeneracies between topologically inequivalent bands. The main signature of 3D gapless topological phases is a 0D Weyl degeneracy, called a Weyl point, which is said to be type-I if the equi-frequency surface at the Weyl frequency is point-like (Fig. 3b) and type-II otherwise (Fig. 3c).

Aside from the different dimensionality, Weyl points are distinct from 2D Dirac points, which are nodal points in 2D momentum space, in some other aspects. The existence of 2D Dirac points is protected by $${\cal{P}}$$$${\cal{T}}$$ symmetry. Thus, they can be easily removed if either $${\cal{T}}$$ or $${\cal{P}}$$ is broken. In contrast, Weyl points exist in systems that lack $${\cal{T}}$$, $${\cal{P}}$$, or both, so Weyl points cannot be eliminated by breaking $${\cal{T}}$$ or $${\cal{P}}$$. This can be understood in that they are monopoles of the Berry curvature in 3D momentum space. Each Weyl point serves as a source or drain of the Berry curvature and carries a quantized topological charge. Thus, two Weyl points always come in pairs with opposite signs of charge and can be created or eliminated only when the Weyl point pairs are generated together or annihilate each other^101^. The topological charge, often called the chirality, of a Weyl point is a topological invariant of 3D gapless phases and can be obtained by integrating the Berry curvature over a small sphere enclosing the Weyl point^101^.

Studies on 3D gapless phases are closely related to Weyl physics. This physics addresses a Weyl particle, which is a solution of the massless Dirac equation, also known as the Weyl equation^107^. The existence of Weyl particles was uncertain for a long time until they were concurrently found in electronic^103,104^ and photonic^108^ systems by three independent groups. These demonstrations were remarkable achievements because no Weyl point had ever been previously identified, while studies on Weyl particles trace back to the early twentieth century^107^. Near Weyl points, electrons (or photons in a photonic system) behave as Weyl particles, which are relativistic particles that have charge but no mass. Another interesting feature of Weyl points is the existence of a Fermi arc between a pair of Weyl points^101,103,109^. The Fermi arc corresponds to topologically protected surface states resilient to perturbations.

Analogously, photonic systems that host Weyl points exhibit topological surface states, which are often called photonic Fermi arcs. The photonic counterparts of these advances in condensed matter physics have been investigated over the past several years. The first achievement in 3D topological photonics was the numerical demonstration of a Weyl point using photonic crystals^108^. Since then, photonic systems that possess Weyl points have been extensively studied and realized in homogeneous media^110,111^, photonic crystals^112–116^, and metamaterials^109,117–119^. Such Weyl systems have exhibited various unprecedented phenomena, such as topological surface states connecting a pair of Weyl points^120^, the quantum anomalous Hall effect^105^, and chiral anomalies^121^.

### Approaches to realize 3D photonic topological phases

In this section, we review 3D photonic topological systems, especially focusing on a variety of approaches to achieve 3D topological phases and related phenomena. For the photonic analog of a 3D topological phase, at least one of ${\cal{T}}$ or ${\cal{P}}$ must be broken^108^. We review the first demonstrations of 3D photonic topological systems that broke either ${\cal{T}}$ or ${\cal{P}}$, or both simultaneously. Then, we discuss ${\cal{T}}$-breaking approaches, followed by ${\cal{P}}$-breaking approaches.

**Fig. 4:**
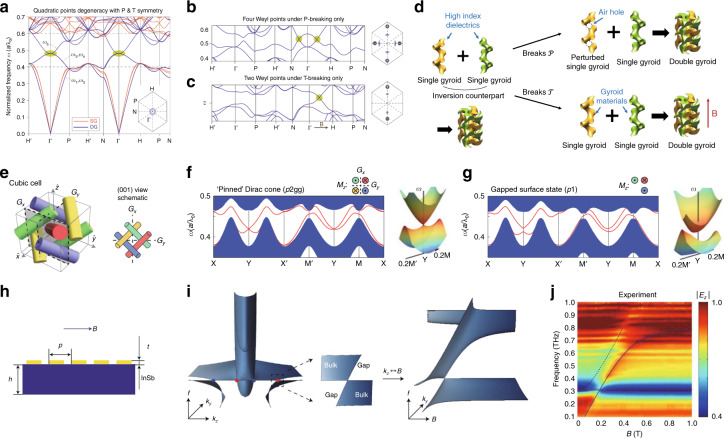
3D photonic topological systems by breaking ${\cal{T}}$ or ${\cal{P}}$. 3D photonic topological systems obtained by (**a**–**d**) breaking ${\cal{T}}$ or ${\cal{P}}$ and (**e**–**j**) breaking ${\cal{T}}$. **a** Band dispersion of single gyroid and double gyroid photonic crystals under both ${\cal{T}}$ and ${\cal{P}}$. **b**, **c** Band dispersion of the double gyroid photonic crystal under **b** ${\cal{P}}$-breaking only and **c** ${\cal{T}}$-breaking only. **a**–**c** Reprinted with permission from Springer Nature: Nature Photonics^108^, Copyright (2013). **d** Schematics of the photonic crystals and symmetry breakings. **e**–**g** Photonic topological insulator protected by glide reflection symmetries. **e**–**g** Reprinted with permission from Springer Nature: Nature Physics^94^, Copyright (2016). **h**–**j** Photonic topological semimetal realized using a magnetized plasma. **h** Schematic of the sample, **i** simulated band dispersion of the magnetized plasma with highlighted Weyl points, and **j** measured reflection spectra under varying magnetic field. The red/blue solid curves and black dashed curve represent bulk and surface states, respectively. **h**–**j** Reprinted with permission from Springer Nature: Nature Physics^110^, Copyright (2019)

The first realization of a 3D photonic topological phase was achieved by breaking the symmetries of double gyroid photonic crystals (Fig. 4a–d)^108^. An array of a single gyroid possesses a wide and complete band gap (Fig. 4a, red lines), whereas a double gyroid photonic crystal, whose unit cell consists of a single gyroid and its inversion counterpart, exhibits a gapless dispersion with a three-fold quadratic nodal point (Fig. 4a, blue lines). The band structure and its topology were studied by breaking symmetries of the double gyroid photonic crystal. The authors first introduced two ${\cal{P}}$-symmetric air spheres into the double gyroids. Under this perturbation that preserves both ${\cal{T}}$ and ${\cal{P}}$, the trivial nodal point is converted into a two-fold nodal ring. However, the nodal ring is topologically trivial as a result of ${\cal{T}}$ and ${\cal{P}}$. Weyl points appear when either ${\cal{T}}$ or ${\cal{P}}$ is broken. Breaking ${\cal{P}}$ by applying the perturbation in which only one air sphere is added without its inversion counterpart leads to four Weyl points (Fig. 4b). This corresponds to the minimal number of Weyl points in ${\cal{T}}$-invariant systems because a Weyl point at ***k*** has a time-reversal pair with the same chirality at −***k*** under ${\cal{T}}$ and another Weyl pair with opposite chirality should exist to neutralize the first pair. In contrast, breaking ${\cal{T}}$ by assuming the gyroid to be a gyroelectric material under a biased magnetic field results in two Weyl points aligned along the magnetic field direction (Fig. 4c). The double gyroid photonic crystal and ${\cal{T}}$-broken and ${\cal{P}}$-broken structures are illustrated in Fig. 4d. Simultaneous breaking of ${\cal{T}}$ and ${\cal{P}}$ by varying their strength has revealed a topological phase transition between pure ${\cal{T}}$-breaking and ${\cal{P}}$-breaking phases. As the amplitude of the magnetic field in a ${\cal{P}}$-broken system increases, a pair of Weyl points with the same chirality moves parallel to the magnetic field direction. When the ${\cal{T}}$-breaking perturbation is strong enough, the Weyl pair finally merges with a Weyl point with opposite chirality, forming a ${\cal{T}}$-dominant topological phase. Since the proposal of the Weyl point, 3D photonic topological systems have been intensively studied using various schemes, as we will see in the following section.

#### By breaking the time-reversal symmetry

Realization of a 3D topological phase by breaking ${\cal{T}}$ provides a concrete platform in which gapless surface states are protected by topology regardless of the spin or valley. Therefore, ${\cal{T}}$-broken topological systems are robust against perturbations that cause spin flipping or valley flipping. However, despite the large number of theoretical investigations and experimental demonstrations of 2D topological phases by breaking ${\cal{T}}$^41–45,122–129^, there exist few examples of 3D photonic topological systems with broken ${\cal{T}}$. Therefore, in this section, we review ${\cal{T}}$-broken 3D photonic topological systems without further classifying them. We discuss theoretical approaches towards 3D gapped and gapless dispersions by using ${\cal{T}}$-broken photonic crystals. Then, homogeneous photonic topological systems based on magnetized plasmas and recent experimental demonstrations are reviewed. Last, we briefly mention 3D photonic topological phases using Floquet’s theorem.

A photonic analog of a 3D strong topological insulator was reported in 2016, in which the surface Dirac cone is protected by crystalline symmetry^94^. The unit structure consists of four identical gyroelectric rods with ${\cal{P}}$ and two glide reflection symmetries (Fig. 4e). When there is no perturbation, the photonic crystal possesses a four-fold Dirac point at the boundary of the BZ; this Dirac point is slightly different from other Dirac points in that it splits into four singlets instead of into two doublets. Breaking of ${\cal{T}}$ by applying alternating magnetizations on the gyroelectric rods while preserving the glide reflection symmetries splits the 3D Dirac point and leads to a single surface Dirac cone (Fig. 4f). The surface Dirac cone is protected by the glide symmetries. Therefore, gapless surface states are observed under the glide symmetries, but the surface states are gapped when the glide symmetries are broken (Fig. 4g).

Breaking of ${\cal{T}}$ can also yield a 3D gapless dispersion by splitting a Dirac point into a pair of Weyl points. In comparison to the ${\cal{P}}$-broken case, Weyl points in ${\cal{T}}$-broken systems have been known to support exotic phenomena such as chiral Majorana edge states and zero modes^44,130^, chiral and gravitational anomalies^118,131^, giant photocurrents^132^, and quantum oscillation phenomena^133^. A tetrahedral photonic crystal composed of anisotropic gyroelectric materials has been proposed to host Weyl points^112^. The tetrahedral photonic crystal with broken ${\cal{P}}$ possesses a three-fold nodal point. Under a magnetic field, the trivial nodal point is lifted, and Weyl points and the consequential topological surface states were numerically demonstrated^112^.

In these photonic crystal-based approaches^108,112^, judiciously designed structures arranged in an adequate lattice are essential. However, Weyl points can also exist in a homogeneous medium: a magnetized plasma, which is a free electron gas under a static magnetic field^111^. If the magnetic field is strong enough that the cyclotron frequency exceeds the plasma frequency, then the longitudinal plasma modes linearly cross the transverse helical modes. This forms two pairs of Weyl points along the magnetic field direction at the plasma frequency. The Weyl points are at the transition between the type-I and type-II states as a result of the flat longitudinal modes at the plasma frequency. This magnetized plasma system has clear advantages over other photonic crystal-based systems: complicated structures are not required, and topological phases can be dynamically controlled by tuning the amplitude and direction of the external magnetic field.

This system was recently experimentally realized by exploiting indium antimonide (InSb), which behaves like a magnetized plasma under a magnetic field in the terahertz regime^110^. The synthetic parameter space (*k*_x_, *k*_y_, *B*) was used instead of (*k*_x_, *k*_y_, *k*_z_) to facilitate the experiment because the strength of the magnetic field acts similarly to *k*_z_ in the Hamiltonian. Therefore, to obtain the band dispersion, the authors fabricated an InSb sample with a metal grating to compensate for the phase mismatch (Fig. 4h) and then measured the reflection spectrum under varying magnetic field. The InSb sample under the magnetic field supports linear band crossing in the terahertz regime (Fig. 4i). The measured reflection spectrum manifests a band crossing at 0.31 THz, which corresponds to a Weyl point, and surface states (Fig. 4j). The topological surface states were further observed under a different measurement setup, where the beam was obliquely injected into the tilted grating on the InSb substrate.

A 3D photonic topological phase can be realized in a slightly different scheme using Floquet’s theorem. A periodically driven 3D network composed of vertically stacked 2D networks can host an ordinary insulating phase, a topological insulating phase, or a topological semimetal phase depending on the coupling strength^134,135^. It is also possible to attain a 3D gapless topological phase possessing Weyl points^77,136^ and a 3D gapped topological phase^78^ in a 2D array of resonators by dynamic modulation.

#### By breaking the inversion symmetry using photonic crystals

An alternative way to achieve 3D photonic topological systems is by breaking ${\cal{P}}$. In this section, we review various numerical and experimental demonstrations of ${\cal{T}}$-invariant 3D photonic topological systems realized by using photonic crystals. The ${\cal{P}}$ of a photonic crystal can be broken by designing a ${\cal{P}}$-broken unit cell. This can be realized by, for example, combining two structures that are not ${\cal{P}}$-symmetric^108^ or breaking the symmetry along the vertical direction^116^. In addition to the double gyroid photonic crystal with air holes, many attempts have been made to achieve a 3D topological phase by using ${\cal{P}}$-broken photonic crystals. An early study used a superlattice of photonic crystals, in which the unit cell was composed of three stacked layers of hexagonal patterns of dielectrics^137^. The authors changed a geometrical parameter of each layer to break ${\cal{P}}$. The resulting unit cell is composed of three unit cells with slightly modified geometries and has a gapless bulk dispersion possessing Weyl points.

**Fig. 5:**
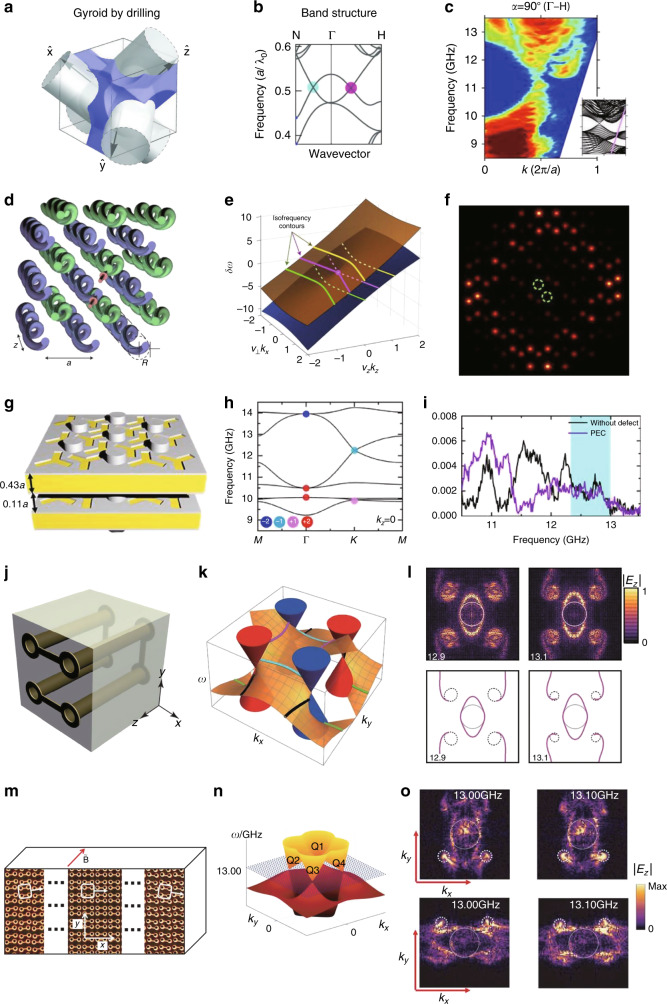
3D photonic topological systems based on photonic crystals. **a**–**c** Experimental observation of type-I Weyl points in a ${\cal{P}}$-broken double gyroid photonic crystal. **a** Schematic of a sample, and **b** simulated band dispersion, and **c** experimentally observed Weyl point based on angle-resolved transmission measurement. **a**–**c** Reprinted/adapted with permission from AAAS^114^, Copyright (2015). **d**–**f** Experimental observation of type-II Weyl points in evanescently coupled waveguides. **d** Schematic of the photonic crystal, **e** simulated band dispersion, and **f** experimentally confirmed conical diffraction obtained by measuring the intensity at the output facet of the fabricated sample. Green circles: position of input waveguides. **d**–**f** Reprinted with permission from Springer Nature: Nature Physics^116^, Copyright (2017). **g**–**i** Photonic crystals possessing multiple Weyl points. **g** Schematic of the photonic crystal, **h** simulated band dispersion, and **i** experimentally measured transmission spectrum, showing no scattering caused by artificially induced defects. **g**–**i** Reprinted from Springer Nature: Nature Communications^113^, Copyright (2016), under Creative Commons Attribution 4.0 International license (CC BY 4.0, https://creativecommons.org/licenses/by/4.0/). **j**–**l** Ideal Weyl photonic crystals and helicoidal surface dispersion. **j** Schematic of a unit cell, **k** numerically, and **l** experimentally obtained helicoidal surface states. **j**–**l** Reprinted/adapted with permission from AAAS^115^, Copyright (2018). **m**–**o** Inhomogeneous topological semimetal designed to host rotating Weyl points and observation of a chiral zero mode. **m** Schematic of a spatially varying photonic crystal, **n** schematic of rotating Weyl points, and **o** experimental demonstration of the chiral zero mode. **m**–**o** Reprinted/adapted with permission from AAAS^118^, Copyright (2019)

The first experimental observation of Weyl points was demonstrated in the microwave range by using double gyroid photonic crystals with broken ${\cal{P}}$.^114^ A single gyroid photonic crystal was fabricated by drilling air holes in a high index dielectric material (Fig. 5a) and then combining the perforated material with its ${\cal{P}}$-broken counterpart. The ${\cal{P}}$-broken double gyroid photonic crystals possess two Weyl points in the microwave regime (Fig. 5b). Because transmission is generally proportional to the bulk density of states, an angle-resolved transmission spectrum was measured to probe the bulk dispersion. The measured spectrum clearly showed a band crossing that corresponds to a type-I Weyl point (Fig. 5c). The four Weyl points with equal frequency in ${\cal{P}}$-broken double gyroid photonic crystals have potential practical applications, such as in angular and frequency selectivity, invisibility cloaking and 3D imaging^138^.

Weyl points can also be experimentally demonstrated by observing conical diffraction, which is a signature of type-II Weyl systems, as implied by the conical equi-frequency curves at the Weyl point. Weyl points have been observed by probing conical diffraction at optical wavelengths^116^. The sample consisted of two interpenetrating square lattices of helical waveguides with a relative phase difference of *π* (Fig. 5d) and was fabricated by femtosecond direct laser writing. Such photonic crystals support type-II Weyl points (Fig. 5e). The intensity on one side was measured during excitation of the opposite side and showed conical diffraction at the Weyl frequency (Fig. 5f). Topological surface states were also observed by exciting the center of the top surface. Weyl points have also been demonstrated using ${\cal{P}}$-broken gyroid photonic crystals coated with layered composite nanometric materials^139^.

Weyl points may carry multiple topological charges higher than unity if an additional spatial symmetry exists that causes overlap of several Weyl points with a single topological charge^113^. To find such multiple Weyl points, the authors used a photonic crystal with a honeycomb lattice, which is known to produce Dirac points in the 2D BZ, and then introduced chiral interlayer coupling (Fig. 5g). Symmetry analysis showed that the double and triple Weyl points are protected by rotational symmetries: C_3_ symmetry and ${\cal{T}}$ for double Weyl points and C_6_ symmetry and ${\cal{T}}$ for triple Weyl points (Fig. 5h). The measured transmission along the samples with and without defects showed similar results, proving that the surface states are not scattered by the defects (Fig. 5i). Soon afterward, multiple Weyl points were theoretically demonstrated in a simple woodpile structure of photonic crystals^140^.

If two Weyl points with opposite signs of chirality merge, the resultant 3D Dirac point has a topological charge of zero due to the annihilation and thus may exist under both ${\cal{T}}$ and ${\cal{P}}$. Similar to the multiple Weyl point cases, the additional degeneracy between two Weyl pairs requires spatial symmetries such as point group symmetry^141^ and nonsymmorphic symmetry^142^. These Dirac points are distinct from other unpaired Dirac points^143^ in that they are paired as a result of the overlap of two Weyl point pairs and carry a nontrivial *Z*_2_ topological phase. Therefore, when the symmetry that ensures overlap of two Weyl pairs breaks, the 3D Dirac point splits into Weyl points^144^.

In a topological semimetal, the dispersion of surface states can be mapped to helicoid Riemann surfaces (Fig. 5k)^145^. However, observation of the helicoidal dispersion of topological surface states has been elusive because of the other bulk bands at the Weyl frequency. It was recently proposed that the helicoidal dispersion may be observable in an ideal Weyl system in which all Weyl points are located at the same frequency and separated from the other bands^115^. To realize this ideal Weyl system, a saddle-shaped metallic coil was designed as a unit structure (Fig. 5j). The metallic coil lacks ${\cal{P}}$ while possessing *D*_2d_ point group symmetry and is periodically arranged in a tetragonal lattice. As a consequence of the absence of other bands at the Weyl frequency, a helicoidal dispersion of the topological surface states could be observed by near-field scanning measurement (Fig. 5l). The existence of the Weyl points was experimentally verified by measuring the angle-resolved transmission.

Recently, an approach for hosting a ${\cal{T}}$-breaking topological phase in a ${\cal{T}}$-invariant system has been reported^118^. To emulate ${\cal{T}}$-breaking, a spatially inhomogeneous structure was designed to induce an artificial magnetic field. Unit cells were designed to support Weyl points that vary spatially in momentum space but are fixed in the frequency axis (Fig. 5m). Starting from the design of a metallic coil that supports four ideal Weyl points^115^, adiabatic variation of two geometric parameters of the unit structure makes the Weyl points rotate in momentum space (Fig. 5n). The rotating Weyl points were confirmed by near-field scanning measurements. The gauge field resulting from the rotating Weyl points makes this ${\cal{T}}$-invariant Weyl system a platform for examining a variety of interesting features that appear under a magnetic field. In the presence of the gauge field, the zeroth Landau level propagates in a chiral way, thereby generating a chiral anomaly. This chiral anomaly was observed by placing a source on one side of the sample and then scanning the near field of the transmitted beam. The Fourier-transformed image of the scanned field at the top (bottom) surface obtained while exciting the sample on the other side dominantly showed positive (negative) Weyl points, as shown in Fig. 5o. This result is consistent with the theoretical prediction that the Landau levels for Weyl points with positive (negative) chirality support a chiral zero mode with a positive (negative) group velocity.

#### By breaking the inversion symmetry using metamaterials

Photonic crystals play a major role in realizing topological photonic bands. However, there is another way to yield topological phases by a radically different mechanism. Metamaterials, which are artificially designed materials to possess extraordinary optical properties, can be exploited to achieve topological phases. Here, the criterion used to distinguish metamaterials from photonic crystals is the validity of the effective medium approximation. Photonic crystals are periodically arranged structures in which the band dispersion is strictly determined by the periodic arrangement and its scale. In contrast, metamaterials that work at the same frequency generally possess much smaller dimensions and can be regarded as homogeneous media. As some readers might sense, the boundaries of this classification are somewhat vague. The operating wavelength is a range, not a specific value, and no strict criterion can be used to determine whether the scale is comparable to or much smaller than the wavelength. In this review, photonic topological systems for which topological phases are attributed to effective parameters are categorized as topological metamaterials.

Whereas the properties of naturally occurring materials are determined by the chemical constituents and their arrangements, the optical properties of metamaterials depend on the artificial unit structure. If the unit structure is much smaller than the wavelength, then light in the metamaterial behaves as if it propagates through a homogeneous medium with effective properties. In other words, the metamaterial can be described as a homogeneous medium with effective parameters even though it is composed of spatially nonuniform structures.

Interestingly, well-tuned effective parameters of metamaterials can induce topological phases. To understand the underlying principle of topological phases, we examine the most general form of the constitutive equation:

2 $$\left( {\begin{array}{*{20}{c}} {\boldsymbol{D}} \\ {\boldsymbol{B}} \end{array}} \right) = \left( {\begin{array}{*{20}{c}} {\hat \varepsilon } & {\hat \zeta } \\ {\hat \xi } & {\hat \mu } \end{array}} \right)\left( {\begin{array}{*{20}{c}} {\boldsymbol{E}} \\ {\boldsymbol{H}} \end{array}} \right)$$

where $\hat \varepsilon$ is the permittivity tensor, $\hat \mu$ is the permeability tensor, and $\hat \zeta$ and $\hat \xi$ are tensors associated with the magnetoelectric coupling. Approaches that use metamaterials can be categorized by the key parameters that produce nontrivial band topology. In this section, we review three different types of topological metamaterials, bianisotropic metamaterials, chiral hyperbolic metamaterials, and Dirac metamaterials, and discuss the key mechanisms of topological phases in each metamaterial using the effective parameters. The constitutive equations of these material responses are summarized in Table 2. Bianisotropic metamaterials obey electromagnetic duality ($\hat \mu = \eta \hat \varepsilon$ for scalar *η*) and have bianisotropy (off-diagonal elements of $\hat \zeta = \hat \xi$), which in combination yield a 3D gapped phase with an even number of surface Dirac cones. Chiral hyperbolic metamaterials simultaneously have hyperbolic (different signs of diagonal elements of $\hat \varepsilon$) and chiral (diagonal elements of $\hat \zeta = - \hat \xi$) properties and support a 3D gapless phase with Weyl points. Dirac metamaterials satisfy electromagnetic duality and have double hyperbolicity (different signs of diagonal elements of $\hat \varepsilon$ and $\hat \mu$ simultaneously), which leads to a 3D gapless phase with Dirac points.

**Fig. 6:**
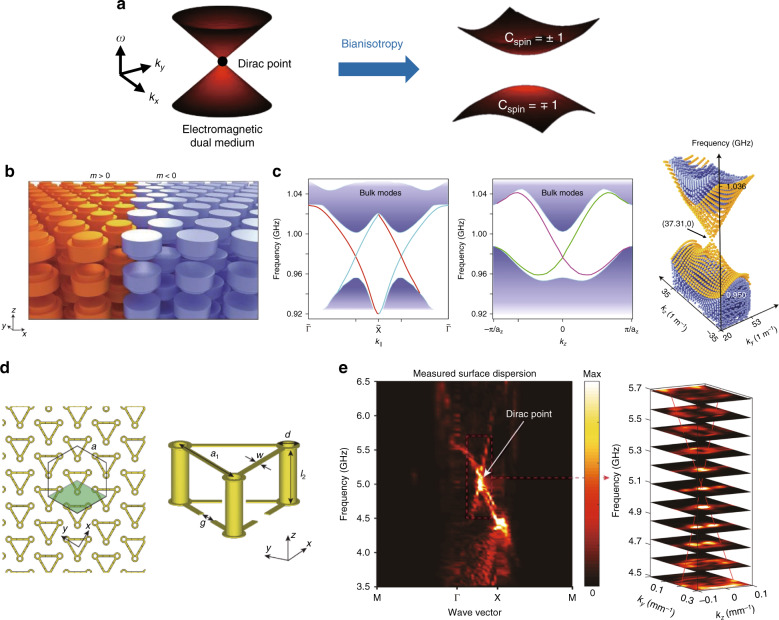
3D gapped photonic topological systems based on bianisotropic metamaterials. **a** Schematic of a bianisotropic metamaterial. A medium with electromagnetic duality has two-fold degenerate modes and a four-fold Dirac point. Adding bianisotropy lifts the Dirac point and opens a gap between bands with different *C*_spin_. **b**, **c** Schematic and dispersion of the bulk (purple) and surface (orange) states of the all-dielectric bianisotropic metamaterial. **b**, **c** Reprinted with permission from Springer Nature: Nature Photonics^99^, Copyright (2016). **d** Schematic, and **e** dispersion of the bulk states of a split-ring resonator-based bianisotropic metamaterial. **d**, **e** Reprinted with permission from Springer Nature: Nature^86^, Copyright (2019)

We first review bianisotropic metamaterials. The realization of electromagnetic duality and bianisotropy to achieve a topological insulating phase was first proposed in a 2D system^8^. When $\hat \mu = \eta \hat \varepsilon$ for scalar *η*, the transverse magnetic (TM) and transverse electric (TE) modes are degenerate due to the electromagnetic duality. To build the photonic counterpart of the Kramers degeneracy, the spin states can be defined as $\psi ^ \pm = E_{\mathrm{z}} \pm H_{\mathrm{z}}$, where *E*_z_ is the out-of-plane electric field of the TM mode, and *H*_z_ is the out-of-plane magnetic field of the TE mode. Then, the two spin states are related as ${\cal{T}}\psi ^ \pm = \psi ^ \mp$. Therefore, in a ${\cal{T}}$-invariant system, the two spin states are doubly degenerate, analogous to Kramers partners. At high symmetric momenta, the bulk states have a gapless dispersion with four-fold Dirac points. Bianisotropy, represented as $\hat \zeta = \hat \xi = i\hat \chi$, is used to produce a band gap. The nonzero off-diagonal components of $\hat \chi$ ($\chi _{{\mathrm{xy}}} = - \chi _{{\mathrm{yx}}} \ne 0$, with all other components $\chi _{{\mathrm{ij}}} = 0$) lift the Dirac point in the 2D BZ and open a topological band gap characterized by a nonzero *C*_spin_ (Fig. 6a). Bianisotropy plays the role of strong spin–orbit coupling in electronic topological insulators and provides a pathway to directly emulate the Kane–Mele Hamiltonian^8^. Spin-polarized edge states with topological protection have been demonstrated numerically using a metamaterial composed of a hexagonal array of bianisotropic rods^8^ and experimentally using a slightly modified design^146^. Bianisotropy has also stimulated studies on topologically nontrivial 2D photonic crystals, called bianisotropic metawaveguides with relaxed design complexity^147–150^.

The topological phase of the aforementioned bianisotropic systems is 2D. However, bianisotropy can also induce a 3D topological insulating phase. A 3D topological gapped system mediated by bianisotropy hosts a full band gap in the whole 3D BZ and topological surface states^99^. A stacked layer of triangular arrays of mirror-symmetry-broken dielectric rods (Fig. 6b) supports a conical dispersion of topological surface states (Fig. 6c) and back-scattering-immune propagation of the surface modes. The broken mirror symmetry makes the in-plane electric and magnetic orbitals couple to each other, which leads to opening of a band gap. The 3D bianisotropic metamaterial is periodic along the out-of-plane direction and therefore has a 3D BZ. The nontrivial 3D topology can be understood as a weak 3D topological insulator, which can be produced by layering arrays of 2D topological systems. Indeed, a bianisotropic metasurface, an array of dielectric bianisotropic unit structures as a 2D version of 3D bianisotropic metamaterials, has been reported to support a 2D topological phase^151^. In contrast to the linear surface Dirac cones in the triangular lattice, quadratic surface dispersions have been demonstrated in the tetragonal lattice of a similar unit structure^100^. Recently, a 3D photonic topological system with a complete and broad band gap was proposed by using a 3D array of metallic split-ring resonators^86^. The authors started with a unit cell composed of six connected split-ring resonators. This mirror-symmetric structure possesses a 3D Dirac point formed by the overlap of two Weyl point pairs. The introduction of bianisotropy by breaking the symmetry along the stacking direction (Fig. 6d) lifts the Dirac point and generates a complete band gap. A gapped bulk dispersion and a surface Dirac cone were observed by Fourier transforming the measured field profile in the bulk and at the domain wall, respectively (Fig. 6e). Robust propagation of surface states was also demonstrated at the interfaces between metamaterials with opposite signs of bianisotropy.

**Fig. 7:**
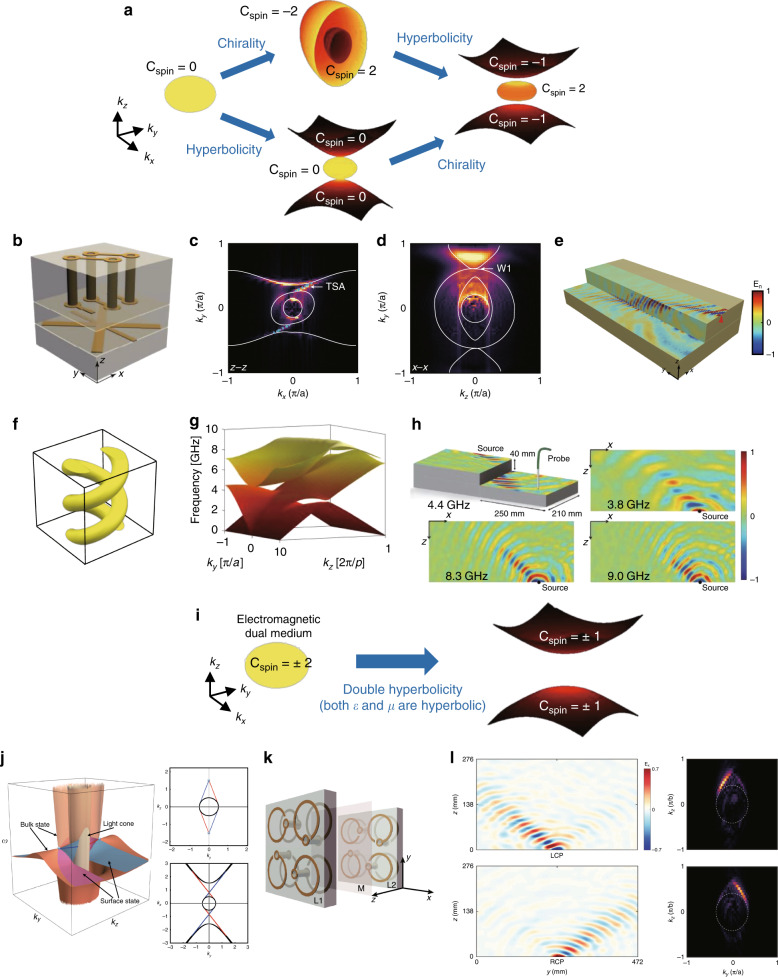
3D gapless photonic topological systems based on metamaterials. (**a**–**h**) Topological systems based on chiral hyperbolic metamaterials. **a** Schematic showing the evolution of the equi-frequency surfaces and Chern numbers of isotropic, chiral, hyperbolic, and chiral hyperbolic media. The equi-frequency surface of an isotropic medium is spherical and topologically trivial. The lack of mirror symmetry for a chiral medium lifts the degeneracy of the two types of circularly polarized light and splits the Chern numbers into ±2. The equi-frequency surface of a hyperbolic medium consists of a sphere and an open hyperboloid, which have no topological phase. The combination of hyperbolicity and chirality generates momentum gaps between topologically inequivalent branches. **b**–**d** First experimental realization of a chiral hyperbolic metamaterial. **b** Unit cell of the chiral hyperbolic metamaterial. **c** Topological surface states and **d** Weyl points in momentum space obtained by Fourier transform of measured electric fields. **e** Experimentally measured topological surface states with suppressed back-scattering. **b**–**e** Reprinted from Springer Nature: Nature Communications^109^, Copyright (2017), under Creative Commons Attribution 4.0 International license (CC BY 4.0, https://creativecommons.org/licenses/by/4.0/). **f**–**h** Broadband chiral hyperbolic metamaterial. **f** Unit cell, **g** simulated band structure with double Weyl points at zero frequency, and **h** broadband topological surface states. **g**, **h** Adapted with permission from ref. ^119^, Copyright (2019) by John Wiley and Sons. (**i**–**l**) Topological systems based on Dirac metamaterials. **i** Schematic of a Dirac metamaterial. A medium with electromagnetic duality has two-fold degenerate modes with *C*_spin_ = ±2. Adding double hyperbolicity maintains the electromagnetic duality while opening the band gap. **j** Bulk and surface state dispersions and light cone of a Dirac metamaterial and equi-frequency curves at (top) and below (bottom) the Dirac point. Reprinted with permission from ref. ^156^, Copyright (2017) by APS. **k** Unit cell and **l** experimentally measured topological surface states of a Dirac metamaterial in real (left) and momentum (right) space. Reprinted with permission from ref. ^157^, Copyright (2019) by APS

A 3D topological phase can also be obtained by combining chiral and hyperbolic properties^152^. Chirality is associated with diagonal elements of $\hat \zeta = - \hat \xi = i\hat \kappa$ and gives rise to distinct optical responses of right circularly polarized and left circularly polarized light. Compared to natural chiral materials, which exhibit exceedingly weak chiral responses, chiral metamaterials are designed to exhibit drastically enhanced chiral light–matter interactions. The origin of the nontrivial topology of the chiral hyperbolic metamaterial is illustrated in Fig. 7a. The equi-frequency surface of an isotropic medium is spherical and topologically trivial. In contrast, the equi-frequency surface of chiral metamaterials is split into two concentric spheres with radii corresponding to the refractive indices of the types of two circularly polarized light. The two types of circularly polarized light possess *C*_spin_ = ±2 due to the spin nature^9^. However, a chiral metamaterial cannot provide a photonic analog of the quantum spin Hall phase because of the closed dispersion. A momentum gap can be introduced by adopting hyperbolicity^152^. Hyperbolic metamaterials refer to metamaterials in which the diagonal components of $\hat \varepsilon$ have opposite signs. Such materials possess equi-frequency contours of a sphere for TE modes and a hyperboloid for TM modes. Although hyperbolic metamaterials are topologically trivial, combining chiral and hyperbolic properties provides a momentum gap between topologically inequivalent modes.

After the first proposal to realize topologically protected surface states in a chiral hyperbolic metamaterial, 3D gapless topological band dispersions and the existence of Weyl points in such materials were reported using effective medium theory^117^. If we define the uniaxial axis of hyperbolicity as the *z*-axis, then introducing anisotropy (*ε*_xx_ ≠ *ε*_yy_) or adding chirality (*k*_xx_ ≠ 0 or *k*_yy_ ≠ 0) into the tangential plane lifts doubly degenerate transverse modes, and each transverse mode forms Weyl points by crossing the longitudinal mode. Weyl points and topological surface states that connect the Weyl points were experimentally observed by near-field scanning measurements in the microwave regime^109^. To simultaneously obtain chiral and hyperbolic properties, a unit cell composed of two layers embedded in a dielectric spacer was employed; the bottom layer was used to realize hyperbolicity, and the top was used to realize chirality (Fig. 7b). The scanned electric field distributions proved that the surface states are immune from back-scattering, and the Fourier-transformed image of the measured electric fields showed topological surface states and Weyl points in momentum space (Fig. 7c–e).

Recently, an array of metallic helices was proposed as a geometrically simple photonic topological platform exhibiting both chiral and hyperbolic properties (Fig. 7f)^119,153^. One advantage of the metallic helix is that the lowest band has topological features. Because of the geometrical chirality, the eigenmodes of the two lowest bands are circularly polarized at long wavelengths. In the zero frequency limit, modes that have opposite signs of *C*_spin_ converge and form a double Weyl point at zero frequency (Fig. 7g). Using this Weyl point at zero frequency, broadband topological surface states with no lower limit were demonstrated in a double-helix array^119^ (Fig. 7h). In addition to Weyl points, a Weyl nodal surface, which is a 2D degeneracy between topologically inequivalent bands, has been demonstrated in a single-helix array^153^. The nodal surface originates from the two-fold screw symmetry and ${\cal{T}}$, while the origin of the topological charge of the nodal surface lies in the chiral and hyperbolic properties of the helices.

A similar but slightly different metamaterial that possesses topological features is a gyromagnetic hyperbolic metamaterial^154,155^. Instead of chirality, the gyromagnetic property can be combined with hyperbolicity to yield the photonic counterpart of the quantum Hall phase by breaking ${\cal{T}}$.

The last type of topological metamaterial is Dirac metamaterials^156^. Magnetoelectric coupling plays no role here: $\hat \zeta = \hat \xi = \hat 0$. Instead, the topological phases of a Dirac metamaterial lie in electromagnetic duality and double hyperbolicity (Fig. 7i). A conventional hyperbolic medium has different signs of diagonal components of $\hat \varepsilon$ and nonmagnetic responses. The eigenmodes of such a medium are linearly polarized; they can be classified as TE and TM. However, if both the $\hat \varepsilon$ and $\hat \mu$ of the medium have different signs of diagonal components and their ratio is fixed ($\hat \varepsilon = \eta \hat \mu$ with scalar *η* > 0), then electromagnetic duality holds, and circularly polarized light becomes the eigenmodes. Additionally, this double hyperbolic medium has a gap in momentum space. A pair of 3D Dirac points, which are four-fold nodal points in 3D momentum space, is observed at the frequency where the two tensors satisfy the electromagnetic duality (Fig. 7j). Each Dirac point is an overlap of two Weyl points with opposite chirality and hence supports double topological surface states connected to the Dirac points. To realize a Dirac metamaterial, a bilayer unit cell composed of eight metallic helices was designed (Fig. 7k). The Dirac point and spin-polarized topological surface states were experimentally demonstrated in the microwave regime^157^ (Fig. 7l). Topological features, such as nonzero *C*_spin_ and topological surface states, have also been observed when the ratio between $\hat \varepsilon$ and $\hat \mu$ is negative^158^ (*η* < 0).

The main advantage of topological metamaterials is that they provide flexible and relaxed conditions in design^119,153^. The topological features of photonic crystals are attributed to Bloch eigenmodes that require translational symmetries. In contrast, topological metamaterials rely on effective medium theory. Although topological metamaterials have been studied under periodic arrangements to calculate the band structure and Chern numbers, translational symmetry is not a necessary condition for effective medium theory. For example, topological features of chiral hyperbolic metamaterials^109,117,119,152^ and Dirac metamaterials^156,157^ have also been confirmed by using effective medium theory and retrieved effective parameters without assuming a periodic arrangement. Therefore, topological phases of topological metamaterials are stable under a disordered arrangement or changes in geometrical parameters as long as the effective parameter conditions are satisfied. Topological phases also remain when the effective parameters are perturbed^159^.

Approaches that use metamaterials have a disadvantage in the deep-subwavelength condition. To support topological phases at a given wavelength, topological metamaterials should be composed of smaller structural units compared to topological photonic crystals. This deep-subwavelength condition may be advantageous in applications such as compact photonic devices, but the design complexity hinders experimental demonstration and practical realization.

## Recent progress in topological photonics

In the previous section, we reviewed numerous attempts to realize 3D topological phases in photonic systems using photonic crystals and metamaterials. In this section, we review and discuss recent progress in topological photonics not covered in the previous sections. We start with stacked topological photonic crystals for layer pseudospin, which were proposed very recently. In addition to the approaches for realizing topological systems, other recent studies have been conducted in a broader context. For example, Weyl degeneracies may have dimensions higher than zero, as opposed to the zero dimension of Weyl points. Additionally, a system possessing a 3D gapped topological phase may support gapless 1D *edge* states instead of gapless 2D surface states. We discuss the cutting edge in topological photonics, especially focusing on six areas: (1) stacked 2D photonic crystals to emulate layer pseudospin, (2) 1D and 2D Weyl degeneracies, (3) unidirectional Maxwellian spin waves, (4) photonic systems with higher-order topological phases, (5) interactions of light with topological phases, and (6) topological photonics oriented to applications.

### Stacked topological photonic crystals for layer pseudospin

Recently, layer pseudospin has emerged as a new degree of freedom. The concept of layer pseudospin was originally studied in 2D materials^160^ and then extended to acoustics^161^ and photonics. In 2019, two independent research groups^162,163^ proposed the use of bilayer photonic crystals to introduce layer pseudospin.

**Fig. 8:**
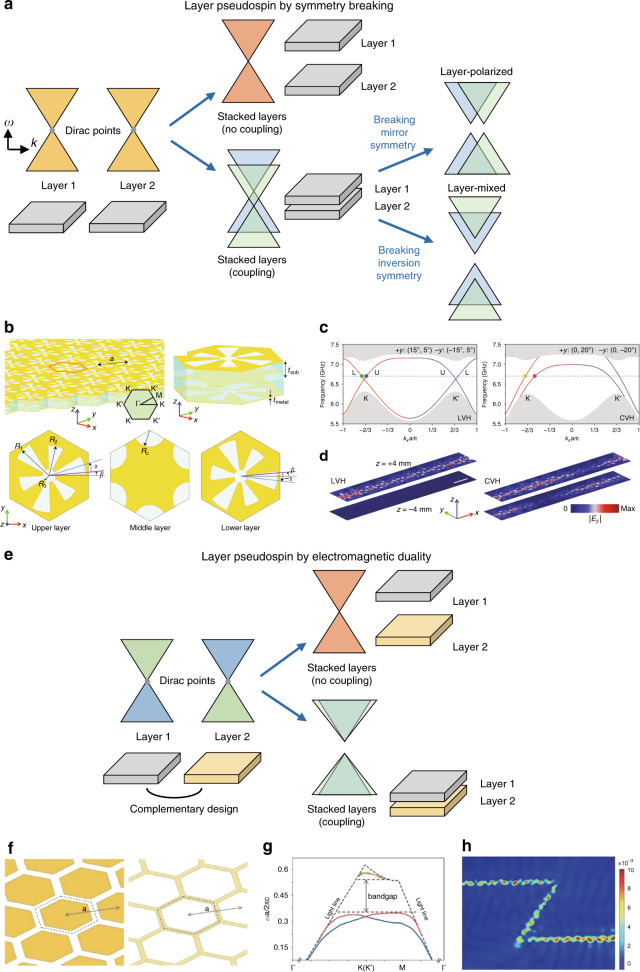
2D photonic topological insulating phases obtained by exploiting layer pseudospin. **a** Schematic of layer pseudospin using symmetry breaking. **b**–**d** 2D photonic topological insulator obtained by breaking symmetries. **b** Unit cell, **c** bulk and surface dispersions, and **d** experimentally measured electric field profiles. **b**–**d** Reprinted with permission from ref. ^162^, Copyright (2019), by John Wiley and Sons. **e** Schematic of layer pseudospin using electromagnetic duality. **f**–**h** 2D photonic topological insulator with electromagnetic duality. **f** Unit cell, **g** bulk dispersion, and **h** experimentally measured electric field profiles. **f**–**h** Adapted from ref. ^164^, Copyright (2019) by John Wiley and Sons, under a Creative Commons Attribution Non-Commercial License 4.0 (CC BY-NC, https://creativecommons.org/licenses/by-nc/4.0/)

One group used dielectric slabs with triangle-shaped air holes arranged in a hexagonal pattern^163^. The layer pseudospin mechanism (Fig. 8a) entails the dispersion of the single slabs, each of which has a Dirac cone. When the separation between two layers is large enough that interlayer coupling is negligible, the dispersion of the bilayer forms doubly degenerate Dirac cones. If interlayer coupling is introduced between two equivalent layers, then the degeneracy is lifted, and the two Dirac cones move oppositely along the frequency axis. The dispersion can be further engineered by using layers with different geometries. When the mirror symmetry is broken while preserving ${\cal{P}}$, the bands are gapped, and two double degeneracies form in the K valley: one above the band gap and one below it. In such cases, fields are localized with counterpropagating power flux in each layer, analogous to spin-orbit coupling in electronics. On the other hand, under a perturbation that lacks ${\cal{P}}$ and preserves mirror symmetry, the bands are gapped but have no degeneracy. Now, the eigenmodes are mixed between two layers that have the same rotation direction of the power flux. These two topological phases are called “layer-polarized” and “layer-mixed” by the authors. Perturbations that simultaneously break mirror symmetry and ${\cal{P}}$ lead to either layer-polarized or layer-mixed topological phases, depending on the relative strengths of the two symmetry breakings.

These two phases were also realized by stacking three layers of patterned metal plates (Fig. 8b)^162^. The first and third layers have slots shaped like ceiling fans, and a topological phase transition occurs by rotating the patterns of the layers. The layer-polarized topological states support two edge states, each localized in each layer, but with opposite group velocities (Fig. 8c, left). This layer-momentum locking shows that the layer pseudospin can be exploited to control edge transport as spin and valley do. However, considering that the edge states localized in two layers can be easily flipped, the layer-polarized phase cannot provide unidirectional propagation. In contrast, in the layer-mixed topological case, the two edge states have the same group velocity sign, positive in the *K* valley and negative in the *K*′ valley (Fig. 8c, right); this arrangement is equivalent to the quantum valley Hall effect. Therefore, the edge states can robustly propagate in the presence of defects as long as intervalley scattering is absent. Edge transport was experimentally demonstrated in the microwave regime by using samples fabricated by circuit-board printing (Fig. 8d)^162^. In these experiments, layer-polarized edge states between media possessing layer-polarized and layer-mixed phases were also observed, suggesting potential in a layer-selected topological delay line.

Recently, photonic crystals composed of two complementary layers were proposed to emulate quantum spin Hall phases. If two complementary layers are placed close to each other, then the interlayer coupling introduces bianisotropy and lifts the Dirac cones (Fig. 8e). Layer pseudospin was recently demonstrated using bilayer photonic crystals composed of a metallic hexagonal patch and its complementary image (Fig. 8f)^164^. Because of the high symmetry, each layer possesses a gapless Dirac dispersion, where the TE and TM modes are degenerate. The two modes are flipped in the complementary layer due to the electromagnetic duality. When the layers are close enough such that interlayer coupling is involved, a band gap appears (Fig. 8g). The bilayer photonic crystals host spin-degenerate states with nonzero *C*_spin_, similar to bianisotropic metamaterials^164^. At the interfaces between bilayer photonic crystals with opposite signs of bianisotropy, topological edge states immune from back-scattering were demonstrated in the microwave range (Fig. 8h)^164^.

### Weyl degeneracies with nonzero dimensions

**Fig. 9:**
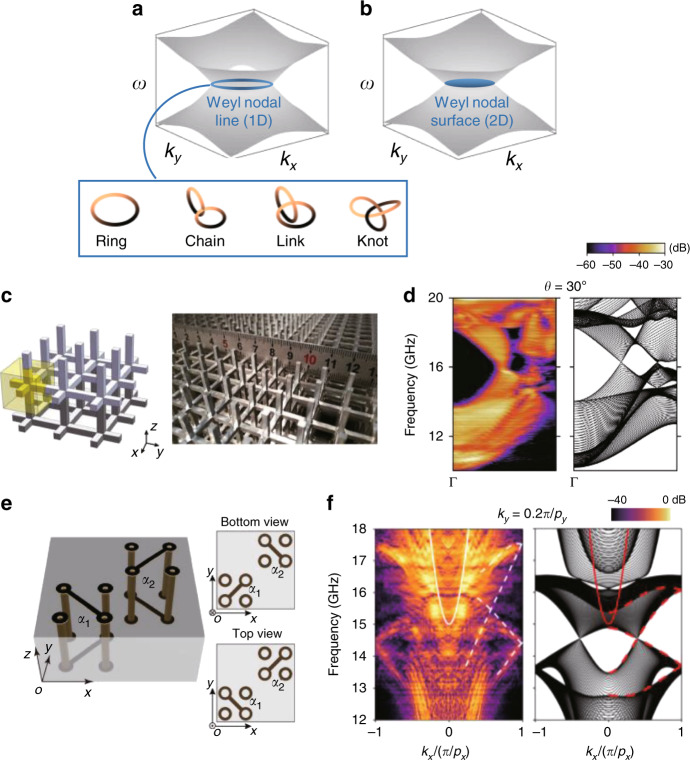
Topological degeneracies in 3D momentum space. **a** Weyl nodal line and four types of nodal line, and **b** Weyl nodal surface. Only the *k*_x_ and *k*_y_ axes among the three momenta are drawn due to the limited dimensions. **c**, **d** Observation of a nodal chain in a metallic-mesh photonic crystal. **c** Schematic and photograph of the fabricated sample. **d** Experimental demonstration of the nodal chain. **c**, **d** Reprinted with permission from Springer Nature: Nature Physics^170^, Copyright (2018). **e**, **f** Observation of an hourglass nodal line. **e** Schematic of the unit cell, and **f** experimental observation of the hourglass nodal line. **e**, **f** Reprinted with permission from ref. ^173^, Copyright (2019) by APS

3D gapless topological phase can possess not only Weyl points but also topological degeneracies that have nonzero dimensions in 3D momentum space. Weyl degeneracies include Weyl nodal lines and Weyl nodal surfaces (Fig. 9a, b). Weyl nodal lines are 1D line degeneracies that can manifest as rings, chains, links, and knots in 3D momentum space^165^. A nodal ring is an ordinary closed loop, while a nodal chain is composed of several nodal rings touching each other. A nodal link and a nodal knot involve links; several nodal rings linked together form a nodal link, and a nodal ring linked with itself is a nodal knot^166^. Photonic nodal rings have been demonstrated using double gyroid photonic crystals^108,114^, a face-centered cubic lattice of a dielectric sphere^167^, and 2D photonic crystal lattices composed of two distinct dielectric cylinders^168^. Experimental observation of a nodal line has been reported using a photonic crystal with ${\cal{T}}$ and two glide symmetries^169^. Although a nodal ring contains topological features, such as a nonzero Berry phase threading the ring, it is not topologically charged and can be split into two Weyl points by breaking ${\cal{T}}$. Nodal chains composed of touching nodal rings, as another type of nodal line, have been experimentally observed in a metallic-mesh photonic crystal (Fig. 9c, d)^170^. However, the nodal lines rely on accidental degeneracies and are therefore prone to be broken or removed by perturbations that even preserve such symmetries as structural variation. In contrast, a nodal line that arises from an hourglass-shaped dispersion of four bands is robust against perturbations that preserve the symmetries^171,172^. An hourglass nodal line was experimentally observed in a photonic crystal possessing three glide mirror symmetries and C_4_ symmetry (Fig. 9e, f)^173^. Recently, nodal links were observed in a biaxial hyperbolic metamaterial^174^.

In contrast to the increasing interest in Weyl nodal lines, studies on Weyl nodal surfaces (Fig. 9b) are scarce in photonics. Nodal surfaces carrying topological charge have been presented in condensed matter physics^175–177^ and acoustics^178,179^, including a recent experimental demonstration. Theoretical predictions suggest that nonsymmorphic symmetries and ${\cal{T}}$ ensure nodal surfaces at the BZ boundary^180^. Weyl nodal surfaces have been numerically demonstrated in photonics using a topological metamaterial possessing such symmetries^153^.

### Unidirectional Maxwellian spin waves

**Fig. 10:**
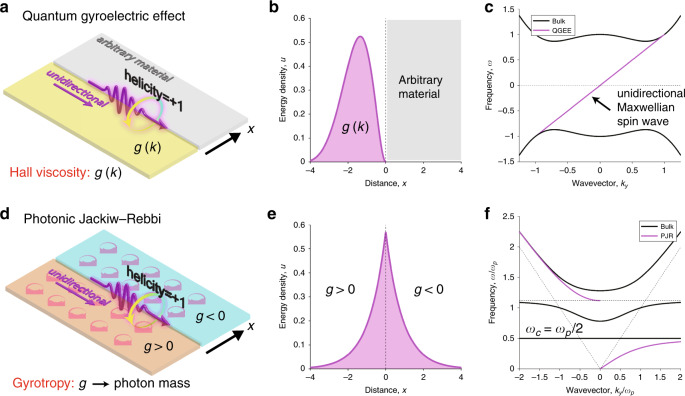
Schematics, energy densities, and dispersions of the quantum gyroelectric effect and photonic Jackiw-Rebbi waves. **a** The quantum gyroelectric phase of matter hosts photonic edge waves, whereas conventional atomic media can only host electronic edge waves. The yellow-colored medium possesses Hall viscosity, which is a necessary condition for observing topological electromagnetic effects in the (2+1)-dimension. **b** The energy density as a function of distance from the interface shows that the photon wavefunction strikingly vanishes on the edge. This disappearance ensures that this topological photonic wave will exist even if the contacting medium is vacuum, a metal, or a semiconductor. No known surface wave has this property. **c** The bulk band gap of a viscous Hall medium is closed by a unidirectional Maxwellian spin wave. **d** Gyrotropy, the high frequency equivalent of the Hall conductivity, was recently shown to be equivalent to the photon mass. Photonic Jackiw–Rebbi waves are analogs of the interface state between a positive mass and a negative mass known in condensed matter physics. **e** The energy density of the photonic Jackiw–Rebbi waves away from the interface shows a single exponential decay. The field is a maximum at the interface and does not vanish like the quantum gyroelectric effect. **f** Dispersion relation of photonic Jackiw–Rebbi waves

Recently, a new phase of matter called a Maxwellian phase was theoretically predicted (Fig. 10a)^51,181–183^. Atomic-scale media can host photonic edge waves analogous to the electronic edge states in condensed matter systems. In the (2 + 1)-dimension, photonic edge waves arise specifically from the Hall viscosity^184^, which is a unique spatially dispersive form of the Hall conductivity. Such edge waves can occur at the interface of a viscous Hall medium and any arbitrary medium, even vacuum (Fig. 10b). These topological waves completely vanish on the edge due to the Hall viscosity and have a biexponential decay away from the interface. The dispersion relation of the bulk and edge shows that a unidirectional Maxwellian spin wave closes the bulk band gap (Fig. 10c)^51,181–183^. The defining property of such an edge wave is helicity quantization, in contrast to conventional classical surface waves, in which the helicity is a classical continuous variable. The theoretical approach known as the Dirac–Maxwell correspondence compares the spin-1 vector fields of light with the spin-1/2 spinor fields in Dirac’s equation^185,186^. Gyrotropy, which is the high-frequency equivalence of the DC Hall coefficient, has been rigorously shown to behave as a mass term for photons^51,181–183^. Therefore, an interface between a positive gyrotropy and a negative gyrotropy can host special spin waves that are unidirectional (Fig. 10d). These waves are photonic analogs of Jackiw–Rebbi waves, which are solutions of the Dirac equation for an interface between a positive mass and a negative mass. These are fundamentally different from the surface plasmon polaritons or magnetic edge plasmons that exist on the interface between media with opposite signs of dielectric constant^45^. The photonic Jackiw–Rebbi waves exponentially decay on both sides of the interface (Fig. 10e). Here, the field has a maximum at the interface and does not vanish like in a viscous Hall medium. Figure 10f shows the dispersion of these photonic Jackiw–Rebbi waves.

### Higher-order photonic topological phase

**Fig. 11:**
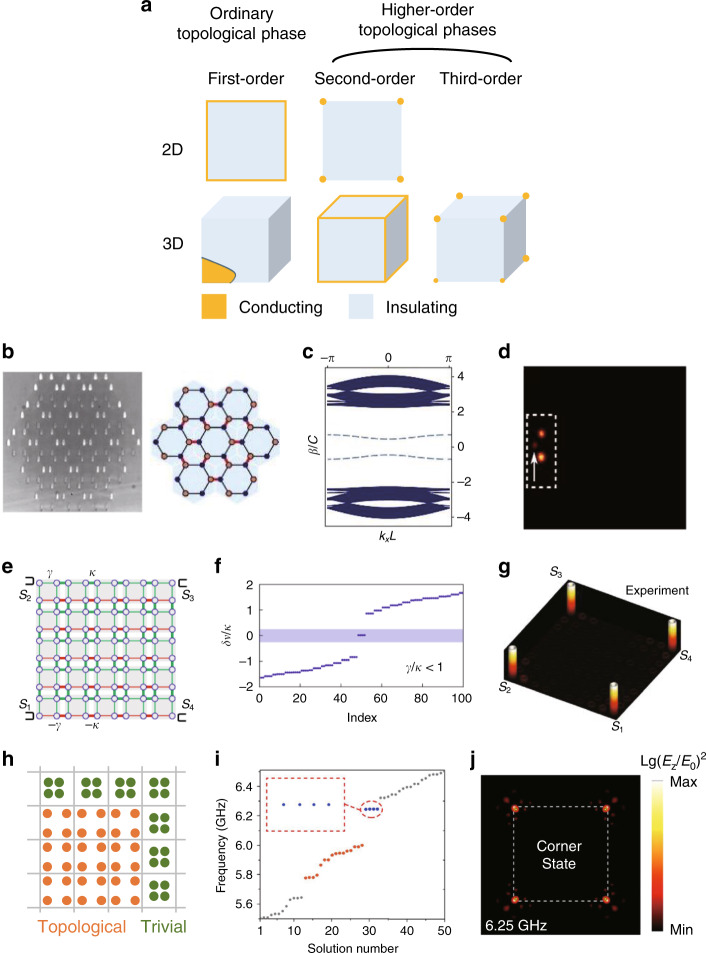
Higher-order topological phases and experimental demonstrations of 2D second-order topological phases. **a** Illustration of first-order, second-order, and third-order topological phases in 2D and 3D systems. Blue: insulating state; yellow: conducting state. **b**–**d** Observation of the topological corner mode in coupled waveguides. **b** Scanning electron microscope image and schematic of the lattice, **c** mid-gap edge states, and **d** experimentally obtained intensity at the output facet to show the topological corner mode. **b**–**d** Reprinted with permission from Springer Nature: Nature Photonics^195^, Copyright (2018). **e**–**g** Topological corner mode in silicon ring resonators with negative coupling. **e** Schematic of the sample, **f** simulated dispersion, and **g** experimentally measured corner mode. **e**–**g** Reprinted with permission from Springer Nature: Nature Photonics^199^, Copyright (2019). **h**–**j** Topological corner mode in the 2D SSH model. **h** Schematic of the 2D SSH lattice for the second-order topological phase. Green: topologically trivial lattice; orange: topologically nontrivial lattice. **i** Simulated dispersion and **j** experimentally measured topological corner modes in the 2D SSH model. **i**, **j** Reprinted with permission from ref. ^203^, Copyright (2019) by APS

The topological insulating phases that we have reviewed thus far have an insulating bulk with gapless boundary modes, which are one dimension lower than the bulk. In other words, a *d*-dimensional topological system possesses (*d* − 1)-dimensional boundary states^187^. It has recently been revealed that there exists a new type of topological phase called a higher-order topological phase that does not obey the traditional bulk-boundary correspondence. Here, topologically nontrivial boundary modes are more than one dimension lower than the bulk. A *d*-dimensional *n*-th order topological insulator has (*d* − 1)−, (*d* − 2)−*, …*, (*d* − *n* + 1)*-*dimensional gapped boundaries, which can be viewed as topological insulators possessing (*d* − *n*)-dimensional gapless boundary states^188^. Examples of such higher-order topological phases include 2D systems with topological corner modes and 3D systems with topological hinge or corner modes (Fig. 11a). Higher-order topological insulators^189–192^ and semimetals^193,194^, which can be obtained by stacking and introducing appropriate interlayer coupling, have been reported in condensed matter physics.

Higher-order topological insulating phases were soon extended to photonics. In 2018, a second-order photonic topological phase was demonstrated using hexagonally arranged evanescently coupled waveguides^195^. The bulk dispersion of the hexagonal lattice was engineered to possess mid-gap edge states by viewing it as a triangular lattice with six hexagonal clusters and arranging them (Fig. 11b)^53^. Whereas a similar structure has been known to support an ordinary first-order topological phase, the mid-gap leads to the existence of topological corner modes (Fig. 11c). 0D modes confined to the corners of the array were observed by measuring the intensity of a diffracted beam at the output interfaces (Fig. 11d). Motivated by the topological corner modes in the breathing kagome lattice in condensed matter physics^193,196^, a similar observation in the visible wavelength regime was conducted using straight waveguides arranged in a breathing kagome lattice^197^. Observation of the second-order photonic topological phase in this breathing kagome waveguide requires a simplified setup that does not require additional waveguides to excite the zero-energy modes. Recently, topological corner modes that originate from long-range interactions beyond nearest-neighbor coupling were observed in a kagome lattice^198^. The long-range interactions were achieved by far-field radiative coupling induced by the transverse mode.

Silicon ring resonators, which have been used to achieve 2D photonic topological phases via asymmetric phases, can also be made to host a second-order photonic topological phase by using a different coupling configuration (Fig. 11e)^199^. The coupling strength and its sign were determined by vertically shifting the links between ring resonators. The ring resonator array has a nonzero quadrupole moment that originates from the negative coupling and the resultant synthetic gauge field. The negative coupling gives rise to topological corner modes in the band gap (Fig. 11f, g).

On the other hand, 2D topological systems with second-order topological phases that do not involve negative coupling also exist. The 2D Su-Schrieffer-Heeger (SSH) model has been proposed to host topological corner modes without requiring negative coupling or a synthetic gauge field^200^. The SSH model^201^, originally defined in 1D, is a dimerized chain, the band topology of which is characterized by the Zak phase. The 1D SSH model is trivial if the intracellular coupling is stronger than the intercellular coupling and nontrivial otherwise. This feature can be straightforwardly generalized to 2D by expanding or shrinking four clusters in a rectangular lattice^82^. When the topologically nontrivial 2D SSH model is enclosed by the trivial 2D model (Fig. 11h), the system supports not only topological edge states but also corner states confined at the corners of the domain boundaries (Fig. 11i)^200^. The corner modes were experimentally observed in the microwave regime by scanning dielectric cylinders arranged in the 2D SSH model (Fig. 11j)^202,203^. Polarization-dependent switching of the topological corner modes was suggested by using plasmonic particles in a 2D SSH lattice under open boundaries^204^. A second-order topological phase in the 2D SSH model was also examined in a microwave circuit^205^. The topological phases in the 2D SSH model are protected by a nontrivial Zak phase, but the quadrupole bulk moment of the model is zero. A 2D SSH model with a nonzero quadrupole moment was recently proposed by breaking ${\cal{T}}$ by means of gyromagnetic materials^206^.

These higher-order photonic topological features have also been found in non-Hermitian systems that can be realized with optical resonators or cold atomic gases^207,208^. Corner modes with topological protection provide a new route to robustly confine light in extremely small mode volumes. This may enable many potential applications such as nanocavities with a high quality factor^209^, low-threshold lasers, and a platform to enhance nonlinearity. To facilitate application in integrated photonics, surface-wave photonic crystals with a broad band gap and topological corner modes were proposed^210^.

### Interactions between light and topological phases

**Fig. 12:**
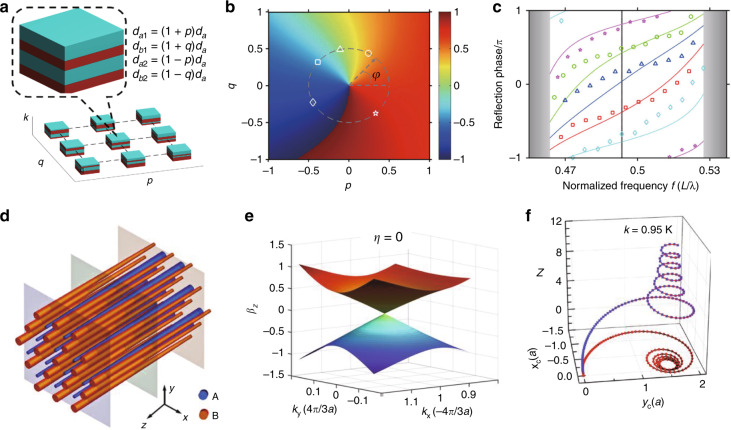
Interactions between light and topological phases. **a**–**c** Phase vortex of the reflected beam observed in a photonic crystal carrying synthetic Weyl points. **a** Schematic of a photonic crystal. **b** Phase of the reflection in parameter space at the Weyl frequency. **c** Simulated and measured reflection phase spectra. (**a**–**c**) Reprinted from ref. 216, Copyright (2017) by APS, under Creative Commons Attribution 4.0 International license (CC BY 4.0, https://creativecommons.org/licenses/by/4.0/). **d** Schematic of a photonic crystal that hosts synthetic Weyl points. **e** Simulated band dispersion with a Weyl point. **f** Trajectory of the wave packet showing helical Zitterbewegung motion. **d**–**f** Reprinted from Springer Nature: Light: Science & Applications^218^, Copyright (2019), under Creative Commons Attribution 4.0 International license (CC BY 4.0, https://creativecommons.org/licenses/by/4.0/)

To date, a variety of approaches to realize photonic topological systems have been reviewed. In this section, we slightly shift our focus and discuss the interplay between light and topological matter. The research discussed here puts more emphasis on determining how photonic topological phases modify previously reported phenomena. The behavior of Weyl particles at the interfaces of two Weyl semimetals has been explored, with a special focus on their valley-dependent transverse shift^211–213^ and scattering^214^. Similarly, the interaction between electromagnetic waves and Weyl media has been studied. At the interface of a Weyl medium and an insulator, a reflected beam undergoes an anomalous shift that maps a half-vortex in momentum space influenced by the topological surface states^215^. The phase vortex of the reflected beam was experimentally verified by using a photonic crystal possessing synthetic Weyl points and a Fabry–Perot interference setup (Fig. 12a–c)^216^. This work suggests a way to detect the topology of Weyl media by measuring beam trajectories.

Furthermore, nontrivial band topology can be used to enhance optical phenomena. It has been demonstrated that the photonic spin Hall effect can be enhanced by the topological edge states of a zigzag array of dielectric particles^217^. More directly, the nonzero Berry curvature near the Weyl points can improve the photonic spin Hall effect. Serving as sources and sinks of the Berry curvature, Weyl points can drastically increase phenomena related to the Berry curvature. Theoretical analysis using a spatially varying waveguide array that supports synthetic Weyl points (Fig. 12d, e) predicted that wave packets propagating near the Weyl points exhibit an enhanced photonic spin Hall effect. Anomalous propagation, called helical Zitterbewegung motion, was also numerically observed along helical trajectories (Fig. 12f)^218^.

The peculiar behavior of photons near a Weyl point also alters the light–matter interactions at both the classical and quantum levels. The conical dispersion at the Weyl frequency decouples the scattering cross-section and wavelength and enables resonant scattering at a desired wavelength^219^. It has been reported that quantum vacuum fluctuations can be engineered by the topology of photonic bands^220^. When a quantum emitter is coupled to a Weyl point at a frequency that matches the transition of the emitter, fractional decay is predicted even when the density states are smooth^221^. Furthermore, the interaction of topological nontrivial systems with nonlinearity has emerged as an interesting field. Topological edge states can be used to increase nonlinear responses such as parametric amplification^220^, optical isolation^222^, Kerr nonlinearity^223^, harmonic generation enhancement^223,224^, and control^225^.

### Topological photonics towards applications

The virtue of topological photonics becomes clearer when we examine applications. The absence of back-scattering modes opens a new route for the implementation of topological photonics in real-world applications. In particular, topological photonics enables dissipation-less transport and thus efficient photonic devices for optical communication. This trait is in contrast to applications based on trivial systems because the energy losses in such systems are strongly affected by device conditions such as defects. Furthermore, whereas topologically protected transport in electronics requires a low temperature, that in photonics operates at room temperature and can be implemented in practical platforms.

Some work has been devoted to developing applications by increasing the practicability of photonic topological systems. Recently, a photonic crystal that supports the quantum valley Hall effect in the terahertz regime was used to transport uncompressed video^226^. The topological boundary modes of the photonic crystal transmitted dynamic signals at high rates, even under a perturbed domain wall. The mass data transfer with robust features proved that photonic topological systems can serve as compact and efficient on-chip devices for communications and data processing. Another paper proposed ultrathin topological photonic crystals that have electrical shielding and can be readily integrated into conventional optical platforms^227^.

For practical applications, a device should give more than one fixed response. Therefore, reconfigurable topological systems that support topological phase transitions that can be manipulated are essential. Dynamic control of topological phase transitions has been achieved using liquid crystals^228^, a phase-change material under temperature variation^229,230^ and transparent conducting oxides under optical pumping^231^. A reconfigurable photonic topological system that supports selective propagation of topological edge modes under mechanical modulation was demonstrated using a bianisotropic photonic crystal^150^. A topological photonic crystal composed of a patterned silicon slab also exhibited tuning of the transmission spectrum under optical pumping^232^.

Some researchers have focused on the practical issues that arise while implementing topological photonics in applications. For example, topological boundary modes should be well coupled with input and outgoing waves. Broadband rerouting of topological edge states excited by propagating plane waves and then released to free space via leaky-wave radiation has been numerically studied by using a magnetized plasma with a patterned plasmonic coating^233^. Whereas a topological system assumes infinitely periodic structures, realistic devices should have finite dimensions. Therefore, studies on these practical limitations, such as the size effect, have been conducted using polariton topological insulators^234^.

Following the currently growing interest in machine learning technology, the design of topological systems by machine learning has been suggested^235–240^. A design methodology, which was originally based on researchers’ intuition and trial-and-error, was developed by training neural networks to estimate topological invariants or find topological band gaps.

## Conclusion and outlook

Despite its short history, topological photonics has been developed surprisingly rapidly. Great success has been achieved in both emulating topological phenomena in photonic systems and finding new exotic features that have no electronic counterpart. The combination of topology and photonics is beneficial for both fields; photonics serves as a tool by which theoretical predictions of topological phenomena can be tested, which has led to the rapid development of topological phases of matter. Topology has also contributed to the growth of photonics by enabling its robust control, even through imperfect devices, and by promoting its implementation in practical applications. The advances in topological photonics have enabled a variety of fascinating phenomena that have not been realized using conventional photonics.

Topological photonics will continue to evolve in the following years, as in the past decade. Classification of the topological phases by their symmetries can provide helpful guidelines for systematically understanding the phases^97,241–244^. Additionally, the scope of the topological phases is becoming diverse, covering nonlinearity^220–225^, non-Hermiticity^245–249^, and dimensions higher than three^250,251^. The scope of topological photonics can be further expanded by combining multidimensional synthetic space and interdimensional mapping^252^ and by including the terms that have been generally ignored, for example, strong coupling of photons^253–256^. Along with previous research on realizing and demonstrating photonic topological systems, studies of how they interact with classical and/or quantum objects and how they alter or enhance the previously reported phenomena may provide surprising insights. In addition to fundamental studies to understand the origin of topology and attempts to realize topological features in many different physical systems, topological photonics has bright prospects in applications, especially when combined with other techniques and interdisciplinary fields.
